# Multi-omics analysis identifies IgG2b class-switching with ALCAM-CD6 co-stimulation in joint-draining lymph nodes during advanced inflammatory-erosive arthritis

**DOI:** 10.3389/fimmu.2023.1237498

**Published:** 2023-08-25

**Authors:** H. Mark Kenney, Javier Rangel-Moreno, Yue Peng, Kiana L. Chen, Jennifer Bruno, Abdul Embong, Elizabeth Pritchett, Jeffrey I. Fox, Enrique Becerril-Villanueva, Armando Gamboa-Domínguez, Sally Quataert, Gowrishankar Muthukrishnan, Ronald W. Wood, Benjamin D. Korman, Jennifer H. Anolik, Lianping Xing, Christopher T. Ritchlin, Edward M. Schwarz, Chia-Lung Wu

**Affiliations:** ^1^ Center for Musculoskeletal Research, University of Rochester Medical Center, Rochester, NY, United States; ^2^ Department of Pathology & Laboratory Medicine, University of Rochester Medical Center, Rochester, NY, United States; ^3^ Department of Medicine, Division of Allergy, Immunology, Rheumatology, University of Rochester Medical Center, Rochester, NY, United States; ^4^ Center for Vaccine Biology and Immunology, University of Rochester Medical Center, Rochester, NY, United States; ^5^ Department of Microbiology and Immunology, University of Rochester Medical Center, Rochester, NY, United States; ^6^ Genomics Research Center, University of Rochester Medical Center, Rochester, NY, United States; ^7^ Psychoimmunology Laboratory, Instituto Nacional de Psiquiatría “Ramón de la Fuente Muñiz”, Mexico City, Mexico; ^8^ Department of Pathology, Instituto Nacional de Ciencias Médicas y Nutrición Salvador Zubirán, Mexico City, Mexico; ^9^ Department of Orthopaedics, University of Rochester Medical Center, Rochester, NY, United States; ^10^ Department of Obstetrics and Gynecology, University of Rochester Medical Center, Rochester, NY, United States; ^11^ Department of Neuroscience, University of Rochester Medical Center, Rochester, NY, United States; ^12^ Department of Urology, University of Rochester Medical Center, Rochester, NY, United States

**Keywords:** spatial transcriptomics, single-cell RNA sequencing, plasma cells, B-cells, arthritis, lymph node, lymphatics

## Abstract

**Introduction:**

Defective lymphatic drainage and translocation of B-cells in inflamed (Bin) joint-draining lymph node sinuses are pathogenic phenomena in patients with severe rheumatoid arthritis (RA). However, the molecular mechanisms underlying this lymphatic dysfunction remain poorly understood. Herein, we utilized multi-omic spatial and single-cell transcriptomics to evaluate altered cellular composition (including lymphatic endothelial cells, macrophages, B-cells, and T-cells) in the joint-draining lymph node sinuses and their associated phenotypic changes and cell-cell interactions during RA development using the tumor necrosis factor transgenic (TNF-Tg) mouse model.

**Methods:**

Popliteal lymph nodes (PLNs) from wild-type (n=10) and TNF-Tg male mice with “Early” (5 to 6-months of age; n=6) and “Advanced” (>8-months of age; n=12) arthritis were harvested and processed for spatial transcriptomics. Single-cell RNA sequencing (scRNAseq) was performed in PLNs from the TNF-Tg cohorts (n=6 PLNs pooled/cohort). PLN histopathology and ELISPOT along with ankle histology and micro-CT were evaluated. Histopathology of human lymph nodes and synovia was performed for clinical correlation.

**Results:**

Advanced PLN sinuses exhibited an increased *Ighg2b/Ighm* expression ratio (Early 0.5 ± 0.1 vs Advanced 1.4 ± 0.5 counts/counts; *p<0.001*) that significantly correlated with reduced talus bone volumes in the afferent ankle (R^2 =^ 0.54, *p<0.001*). Integration of single-cell and spatial transcriptomics revealed the increased IgG2b^+^ plasma cells localized in MARCO^+^ peri-follicular medullary sinuses. A concomitant decreased *Fth1* expression (Early 2.5 ± 0.74 vs Advanced 1.0 ± 0.50 counts, *p<0.001*) within Advanced PLN sinuses was associated with accumulation of iron-laden Prussian blue positive macrophages in lymph nodes and synovium of Advanced TNF-Tg mice, and further validated in RA clinical samples. T-cells were increased 8-fold in Advanced PLNs, and bioinformatic pathway assessment identified the interaction between ALCAM^+^ macrophages and CD6^+^ T-cells as a plausible co-stimulatory mechanism to promote IgG2b class-switching.

**Discussion:**

Collectively, these data support a model of flare in chronic TNF-induced arthritis in which loss of lymphatic flow through affected joint-draining lymph nodes facilitates the interaction between effluxing macrophages and T-cells via ALCAM-CD6 co-stimulation, initiating IgG2b class-switching and plasma cell differentiation of the expanded Bin population. Future work is warranted to investigate immunoglobulin clonality and potential autoimmune consequences, as well as the efficacy of anti-CD6 therapy to prevent these pathogenic events.

## Introduction

Rheumatoid arthritis (RA) is a complex autoimmune disease that is also driven by innate immune pathology (e.g., overproduction of tumor necrosis factor; TNF), which together contribute to joint inflammation, pain, and connective tissue destruction. In fact, the autoimmune nature of RA is demonstrated through multiple pathophysiologic features, including the utility of diagnostic autoantibodies (e.g., anti-citrullinated protein antibodies; ACPAs) (1) and coinciding B/T-cell dysregulation (2, 3). However, major unanswered questions in RA pathogenesis remain, such as: 1) how does seronegative RA arise and progress in the absence of known autoantibodies and 2) how does chronic “sterile” inflammation in affected joints contribute to the development of autoantibodies? Towards answers to these important open questions, it is known that RA progression involves lymphatic dysfunction and alterations in joint-draining lymph nodes (4). Specifically, RA patients exhibit reduced lymphatic drainage from inflamed joints (5, 6), and power doppler ultrasound studies (PDUS) also demonstrate dynamic intra-parenchymal changes and vascular flow modulation of draining lymph nodes linked to the underlying pathologic process (7, 8). Interestingly, RA joint-draining lymph nodes contain large numbers of quiescent, polyclonal CD23^+^/CD21^hi^ B-cells in inflamed nodes (Bin cells), but their pathogenic role remains unknown (9, 10).

To elucidate the aforementioned RA pathology and to develop more effective interventions, we characterized the natural history of inflammatory-erosive arthritis in TNF-transgenic (Tg) mice, which is a well-established autoantibody independent model of RA (9). Interestingly, the 3647 TNF-Tg mouse line, which contains one copy of the transgene, recapitulates all the draining lymph node features and lymphatic dysfunction observed in RA patients (4). During “Early” arthritis, the ankle draining popliteal lymph nodes (PLNs) demonstrate marked volume expansion and increased blood flow (4). PLN expansion persists to ~8-months of age in male TNF-Tg mice, followed by stochastic and asymmetric collapse of PLNs, associated with reduced volume and blood flow, and rapid onset of “Advanced” arthritis with severe synovitis and bone erosion (4). Of note, female TNF-Tg mice die from cardiopulmonary disease by ~5 months of age (11), which limits the potential investigation of the chronic lymphatic phenotypes and mechanisms.

Histologic assessments of PLNs from TNF-Tg mice with Early and Advanced arthritis provide insight into the cellular mechanisms mediating these dynamic lymph node changes (4, 12, 13). In Early arthritis, the CD23^+^/CD21^hi^ Bin cells accumulate in PLN follicles while afferent CXCL13-expressing macrophages egress through the expanded sinuses. Collapsed PLNs in Advanced arthritis are characterized by translocated Bin cells into the sinuses (4, 12, 13), presumably via a CXCL13 gradient produced by stagnant macrophages that cannot egress due to the lack of lymphatic vessel contractions and passive flow (4). Bin cell “clogging” of efferent lymphatics is hypothesized to exacerbate inflammatory-erosive arthritis, as their removal with anti-CD20 B-cell depletion therapy in TNF-Tg mice restores lymphatic flow and ameliorates Advanced arthritis (4, 12, 14).

Recent attempts to reveal the mechanisms responsible for lymphatic dysfunction and Bin cell accumulation and translocation in PLN sinuses during Early vs Advanced arthritis in TNF-Tg mice utilized various bulk-tissue approaches (i.e., bulk RNA sequencing, flow cytometry and *ex vivo* cultures), which lack the resolution to identify molecular changes related to arthritic severity at the single-cell level (4). Furthermore, these experiments are also limited by contamination of follicular B-cells with the inability to differentially assess B-cell phenotypes based on spatial location within the PLN sinuses. Single-cell RNA sequencing (scRNAseq) and spatial transcriptomic technologies overcome these limitations by providing a detailed topographic view of cell-specific identity, localization, and predictive cell-cell interactions via ligand-receptor expression, which has transformed our understanding of RA etiology and pathogenesis (15, 16). Thus, we applied these approaches to 1) examine if arthritic severity of afferent ankle joints correlates with unique phenotypes of B-cells within PLN sinuses of TNF-Tg mice and 2) identify spatial relations and potential co-stimulatory pathways among various immune cells that contribute to the initiation of PLN immunopathology and autoimmunity.

## Materials and methods

### Mouse models

All animal experiments were approved by the University Committee for Animal Resources at the University of Rochester. Only male wild-type (WT) and TNF-Tg (C57BL/6 background) mice were used for this study due to the accelerated mortality of females (11). The 3647-line of TNF-Tg mice (17), originally obtained from Dr. George Kollias, have been maintained at the University of Rochester. Individual limbs were used as the experimental unit based on the asymmetry of TNF-Tg arthritis (5, 11–14, 18, 19). A total of 41 mice were used in this study, and quantitative metrics were evaluated with a minimum of n = 6 experimental units and n = 3 for qualitative histology, similar to previous studies (5, 20). Specific sample sizes for each experiment are provided in the relevant sections below. All mice were used for the study except three Advanced ankle joints were excluded from histologic assessment due to insufficient decalcification; otherwise, no mice or samples were excluded from the study. Animals were allocated into experimental groups based on age. Previously, we demonstrated that the joint disease in TNF-Tg mice can be defined as “Early” by mild synovitis and bone erosions associated with PLN expansion (increased volume and blood flow), and intact PLV contraction frequency and clearance in male mice at 5- to 6-months of age. On the other hand, “Advanced” joint disease occurs concomitant with PLN collapse (reduced volume and blood flow) and failure of PLV contractility that is commonly observed in male mice starting at 8-months of age (4, 5, 12–14, 18, 21). All mice were housed in the same traditional caging environment and exposed to a 12-hour light/dark cycle to limit potential confounders. TNF-Tg mice were monitored weekly for failure to thrive and were provided diet supplementation with Nutra-Gel (2-ounce cups, Bio-Serv). There were no unexpected adverse events for the mice allocated to this study. Investigators performing histologic stains and histomorphometric analysis were blinded to the group allocation of the samples while conducting the experiment.

### Spatial transcriptomics tissue optimization

PLNs from WT males (n=2 mice, 4 PLNs) were harvested, embedded in optimal cutting temperature (OCT) compound (Cat# 4583; Sakura, Osaka, Japan), and frozen in liquid nitrogen. Six tissue sections of 10μm were placed within individual capture areas of a 10X Genomics Optimization slide and stored at -80°C. The tissue sections were then fixed in cold methanol and stained with H&E according to the Visium Methanol Fixation and H&E Staining guide (10X Genomics). An Olympus VS120 slide scanner was used for brightfield imaging of the H&E-stained sections. To evaluate the optimal permeabilization time, each capture area was permeabilized for a different time period (3, 6, 12, 18, 24, or 30 minutes) followed by reverse transcription with fluorescent nucleotides to label cDNA. Tissue sections were then removed, and the slide was imaged again using the Olympus VS120 slide scanner to evaluate fluorescence with the TRITC filter. The optimal permeabilization time was determined to be 12-minutes by balancing the brightest fluorescent signal intensity with the least amount of diffusion (**Supplementary Figure 1**), and the 12-minute permeabilization time was then applied to the sections of the gene expression slide.

### Spatial transcriptomics

PLNs from 5-6-month-old WT (n=5 mice, 10 PLNs), 5-6-month-old TNF-Tg (Early arthritis; n=3 mice, 6 PLNs), and >8-month-old TNF-Tg (Advanced arthritis; n=6 mice, 12 PLNs, 2 replicate tissue blocks of 6 PLNs each) mice were harvested, embedded in OCT, and frozen in liquid nitrogen. The PLNs were patterned in the block such that corresponding animal sample ID could be identified for post-hoc analysis. To evaluate RNA integrity numbers (RIN) for quality control, ten-consecutive 10μm sections were collected with a minimum RIN value of 9.6 for the gene expression samples (**Supplementary Table 1**). A 10μm tissue section was then placed within the 4 capture areas of a 10X Genomics Gene Expression slide and stored at -80°C. The tissue was then fixed with cold methanol and H&E stained based on the Visium Methanol Fixation and H&E Staining guide (10X Genomics). An Olympus VS120 slide scanner was used for brightfield imaging of the slide at 200X, and .tif files were saved for downstream analysis in the SpaceRanger software. The tissue sections were then permeabilized for 12-minutes to release poly-adenylated mRNAs, which were captured by primers on the slide. The Visium Spatial Gene Expression Kit was then used for reverse transcription to produce full-length cDNA. The cDNA was assessed by KAPA qPCR to optimize the cycle numbers for cDNA amplification. Following amplification, the cDNA was purified using SPRISelect beads (Agilent, Santa Clara, CA). The Visium Library Construction Kit (10X Genomics) was then used to generate Illumina-compatible sequencing libraries with approximately 25% of the total cDNA yield. cDNA amplicon size was optimized, and indexed sequencing libraries were constructed by End Repair, A-tailing, Adaptor Ligation, and PCR. The NovaSeq 6000 sequencer (Illumina, San Diego, CA) was used to sequence the amplified libraries on an SP flow cell of 28x10x10x90 to obtain approximately 50,000 reads per spot for each capture area covered by tissue, according to the sequencing requirements in the Visium Spatial Gene Expression manual.

### Spatial transcriptomics analysis

The filtered feature matrices were imported and analyzed using the Seurat packages (v4.0.3) in RStudio (v1.2.1335; R v4.1.1) (22). The data was then processed according to instructions of the Seurat R package, where a shared nearest neighbor (SNN) clustering algorithm was used to embed the data as a Uniform Manifold Approximation and Projection (UMAP). Spot clusters were defined using resolution = 0.5, and both unsupervised and minimally supervised clustering (i.e., combination of phenotypically associated clusters) were performed based on their genetic expression profiles and spatial location in the PLN. The different capture areas were merged to define relative spot counts of related clusters. Two replicates of spatial transcriptomic datasets from Advanced arthritis (n=6 mice, 12 PLNs, 6 PLNs/replicate) were also integrated by comparable gene expression in each spot (**Supplementary Figure 2**). For analysis of individual PLNs, Loupe Browser (v5.0.1, 10X Genomics) was used to manually select PLN-specific spots. The selected PLN spots were exported as separate csv files and imported into Seurat for downstream analysis. The *AverageExpression* function in Seurat was also used to quantify the expression of particular genes within specific spatial clusters in the individual PLNs. Note that the spatial transcriptomics spots are not at single-cell resolution, but instead represent 55μm diameter spots for spatially-mapped bulk-sequencing of all cells within a particular spot. Thus, to estimate IgG expression by single B-cells within the sinuses by spatial transcriptomics, we normalized to *Ighm*, which is highly expressed by all B-cells via scRNAseq (**Supplementary Figure 6**).

### scRNAseq and bioinformatic analysis

PLNs from TNF-Tg mice with Early and Advanced arthritis (n = 3 mice pooled per group) were harvested and surrounding tissue was removed. The PLNs were incubated in 10% fetal bovine serum (FBS) in Dulbecco’s modified Eagle’s medium (DMEM) + GlutaMAX (Gibco, Cat# 10566-016), followed by mechanical disruption of tissue placed in a petri dish with 1.5mL of Accumax (Innovative Cell Technologies, Cat# AM-105) with a scalpel. Minced tissue in Accumax was then transferred into a 1.5mL Eppendorf tube and rotated for 1-hour at room-temperature. After the digestion, single cells were passed through a 70μm MACS SmartStrainer (Miltenyi Biotec, Cat# 130-098-462) that was pre-wet with 3mL of 20% FBS in PBS into a 15mL conical tube on ice. 1mL of 20% FBS was used to clean the Eppendorf tube then similarly passed through the cell strainer to quench the enzymatic reaction. All samples for a particular group were pooled together, pelleted at 300g and incubated at 4°C for 10-min, and resuspended in 2% FBS in PBS. The cell suspension was stained with Hoechst 33342 (exclude red blood cells; NucBlue Live ReadyProbes, ThermoFisher Scientific, Cat# R37605) and Sytox Green (exclude dead cells; NucGreen Dead 488 ReadyProbes, ThermoFisher Scientific, Cat# R37109). An aliquot was set aside as an unstained control. Live cells were then sorted on a BD FACSAria II using a 100μm nozzle (20psi sheath pressure) into a 2mL Eppendorf collection tube that had been filled completely overnight with 100% FBS for coating, and replaced with 200μL of fresh 2% FBS collection media prior to sorting. A total of 100,000 Hoechst^+^/Sytox^-^ events were collected for each group and processed for single-cell RNA sequencing by the Genomics Research Center at the University of Rochester Medical Center. The cells were sequenced using Illumina’s NovaSeq 6000.

The datasets were analyzed according to the instructions of the Seurat package in R with unsupervised SNN clustering performed at resolution = 0.5. For the analysis, genes were removed if the gene was only expressed by < 3 cells, and low-quality cells were also removed if the cell exhibited < 200 genes. Differential gene expression analysis was evaluated and used for annotation of each cluster. To determine potential cell-cell interactions, the NicheNet R package (v1.0.0) (23) was applied to the Seurat objects using commands available through SeuratWrappers (v0.3.0), and only *bona fide* interaction potential was evaluated (i.e., supported by literature). A published scRNAseq dataset of lymphatic endothelial cells (LECs) [GSE145121 (24)] was integrated with our spatial transcriptomics datasets to assess specific LEC populations. For identification of LECs, we used the published scRNAseq dataset that specifically enriched for LECs through flow sorting, while hematopoietic cells predominated in our total live cell collections used in this study. The published data were reclustered to identify the LEC subpopulations. Then, overlap of gene expression between the spatial spots (not single-cell level) and the single-cell data allowed us to enhance the cellular resolution by generating a cell phenotype prediction score of cell localization to unique spatial spots. Analysis was performed according to the instructions of the “Analysis, visualization, and integration of spatial datasets with Seurat” vignette provided by the Satija laboratory.

In the published scRNAseq dataset by Xiang et al. [GSE145121 (24)], the investigators utilized a rigorous enrichment for lymph node LECs. Briefly, the authors performed magnetic depletion of CD45^+^ hematopoietic cells followed by flow sorting of LECs (CD31^+^/PDPN^+^ & CD45^-^/EpCAM^-^/TER119^-^/CD11a^-^/CD11b^-^) for downstream scRNAseq (10X Genomics). The investigators also performed additional corresponding scRNAseq experiments (SmartSeq2) with LECs sorted from *Prox1-GFP* mice (C57BL/6 background) with magnetic depletion of CD45^+^ hematopoietic cells along with flow sorting for live LECs (CD31^+^/PDPN^+^ & CD45^-^/TER119^-^/CD11b^-^). For the sequencing, the combination of both droplet- (10X Genomics) and plate-based (SmartSeq2) approaches provided the dual benefit of high-throughput (droplet) and increased sequencing depth (plate) to elucidate the distinct LEC subsets (25). From the datasets provided (10X Genomics), we specifically utilized the scRNAseq data from C57BL/6 mice, corresponding with the genetic background of the TNF-Tg strain. In addition, the published study utilized peripheral lymph nodes of the extremities, pooling together multiple brachial, axillary, and inguinal lymph nodes, which would exhibit comparable function and lymphatic drainage of the limbs as PLNs.

### Micro-CT data collection and analysis

*Ex-vivo* micro-CT on the hind paws was performed to evaluate and correlate the severity of ankle arthritis with gene expression in the efferent PLN. Micro-CT datasets were acquired using a VivaCT 40 (Scanco Medical, Bassersdorf, Switzerland) with the following imaging parameters: 55kV, 145μA, 300ms integration time, 2048 x 2048 pixels, 1000 projections over 180°, resolution 17.5μm isotropic voxels. The DICOM files were then imported into Amira software (v2020.2, ThermoFisher Scientific, FEI, Hillsboro, OR, USA) and talus bone volumes were measured, as previously described (18, 26).

### Histology, immunostaining, and image analysis

PLNs from additional Early and Advanced TNF-Tg mice (n = 3 per group) were harvested, embedded in paraffin, and used for histologic analysis, while the ankle joints from the mice used for spatial transcriptomics were utilized. Due to inefficient decalcification and deficient soft-tissue removal, ankle joints from n = 3 Advanced mice were excluded, and the scanned ankle joints from the mouse with Advanced arthritis used for PLN 3D rendering was used for histology. PLNs and ankle joints were fixed in 10% neutral buffered formalin (NBF) for 2h and 3-days, respectively. PLNs were then embedded in paraffin, while ankles were decalcified in Webb-Jee 14% EDTA solution for 1-week prior to paraffin-embedding. Tissue sections were then collected at 5μm. The human synovial samples were processed according to the Accelerating Medicines Partnership (AMP) Program’s standard operating procedures (15, 27–30). Human LNs from patients with RA or those with signs of thyroid cancer were obtained from archived tissues in the National Institute of Medical Sciences and Nutrition “Salvador Zubiran”.

For Prussian Blue staining, both frozen sections from the spatial transcriptomics tissue blocks and paraffin-embedded sections were evaluated. The frozen PLN spatial transcriptomics sections were used for quantification of iron-laden cells, while the PLN paraffin-embedded sections were used for representative images of the Prussian blue stain. Frozen sections were first fixed in chilled 10% NBF for 10-minutes then stained with Prussian Blue and counterstained with Nuclear Fast Red. The paraffin-embedded sections were dewaxed and rehydrated before the staining process. Paraffin-embedded ankle sections were also stained with Prussian Blue. Each slide was then imaged using a VS120 Slide Scanner, and .vsi files were analyzed using semi-automated segmentation workflows in Visiopharm (v2021.07; Horsholm, Denmark).

For immunofluorescence staining, slides were incubated at 60°C overnight to melt the paraffin and hydrated by immersion in xylenes, graded alcohols and water. Antigen retrieval was performed by boiling slides in a Coplin jar filled with Citrate-Based Antigen Unmasking Solution (1:100 dilution; Vector Laboratories, Cat# H-3300). After cooling to room temperature, non-specific staining was prevented by incubating tissues with normal donkey serum (NDS; Jackson ImmunoResearch, Cat# 017-000-121) in TBS (Bio-Rad, Cat# 1706435)/0.3% Triton X-100 (Millipore Sigma, Cat# X100) for 40-min at room temperature. The primary antibodies were then diluted in the NDS solution and applied at 4°C overnight. After three PBS washes, secondary antibodies were similarly diluted in the NDS solution and incubated on the slides at room temperature for 1-hour. The slides were then washed with PBS three times and mounted using one drop of NucBlue Live ReadyProbes (Hoechst 33342) and ProLong Gold Antifade Mountant (ThermoFisher Scientific, Cat# P36930). Finally, the slides were imaged using a VS120 Slide Scanner or a Zeiss Axioplan Microscope.

Antibodies and dilutions for immunofluorescent staining: Rabbit anti-mouse CD6 (ThermoFisher, Cat# MA5-29680, RRID: AB_2785505, 1:50), rat anti-mouse F4/80 (BioRad, Cat# MCA497R, RRID : AB_323279, 1:50), rabbit anti-mouse MARCO (Abcam, Cat# ab239369, 1:100), Alexa Fluor 555 goat anti-mouse IgG2b cross-adsorbed (ThermoFisher Scientific, Cat# A-21147, RRID : AB_2535783, 1:50), APC rat anti-mouse CD45R/B220 (BD Biosciences, Cat# 553092, RRID : AB_398531, 1:100), goat anti-mouse ALCAM (CD166; ThermoFisher, Cat# PA5-47083, RRID : AB_2607383, 1:100), Cy3 goat anti-mouse IgM (Jackson ImmunoResearch Laboratories, Cat# 115-165-020, RRID : AB_2338683, 1:200), FITC donkey anti-mouse IgG (Jackson ImmunoResearch Laboratories, Cat# 715-095-150, RRID : AB_2340792, 1:200), goat anti-proliferating cell nuclear antigen (PCNA; Santa Cruz Biotechnology, Cat# sc-9857, RRID : AB_2160372, 1:50), FITC Peanut Agglutinin (PNA; Millipore Sigma, L7381-1MG, 1:50), goat anti-mouse peripheral node addressin (PNAd; BD Biosciences, Cat#553863, RRID : AB_395099, 1:50), rabbit anti-mouse LYVE1 (Acris Antibodies, Cat# DP3513, RRID : AB_1004776, 1:50), AlexaFluor 568 donkey anti-goat IgG (ThermoFisher Scientific, Cat# A-11057, RRID : AB_142581, 1:200), AlexaFluor 647 F(ab`)_2_ fragment donkey anti-rat IgG (Jackson ImmunoResearch, Cat# 712-606-153, RRID : AB_2340696, 1:200), and AlexaFluor 647 donkey anti-rabbit IgG (Abcam, Cat# ab150075, RRID : AB_2752244, 1:400).

### Human lymph node samples

Human lymph node samples were provided by Drs. Enrique Becerril-Villanueva and Armando Gamboa-Dominguez from controls with thyroid cancer negative for lymph node metastasis (n=2) and RA autopsy subjects (n=2). Collection of specimens from RA autopsy subjects was conducted with written and signed consent from their family members in accordance with the Declaration of Helsinki and after approval from the Ethical Committee of the National Institute of Medical Sciences and Nutrition “Salvador Zubirán”. Control lymph node specimens were collected based on approved protocols by the Committee of Human Research. Analysis of deidentified tissue specimens was performed according to protocols approved by the University of Rochester Institutional Review Board. Patients met the ACR/EULAR criteria for RA (31, 32) or received a clinical diagnosis of RA from an experienced rheumatologist.

### Human synovial samples

Human synovial samples were provided by Dr. Jennifer Anolik from arthroplasty or synovectomy tissues (RSRB00055411) collected using Accelerating Medicines Partnership (AMP) standard operating procedures (15, 27–30). Prussian blue staining was evaluated in synovial samples from patients with osteoarthritis (OA, n = 3) and RA (n = 3) [fulfilling 2010 ACR/EULAR classification criteria (32)]. OA samples had limited Prussian Blue staining and the region containing the maximum iron-laden cells is depicted. All of the RA samples were derived from CCP^+^ subjects. Two RA samples looked similar to OA, with limited to no iron-laden cells. One of these RA samples did not have a histology pathotype (30) assigned and lacked information about treatments. The second RA sample had a lymphoid pathotype, and the patient received infliximab. The RA sample depicted exhibited regions of abundant iron-laden cells. This synovectomy tissue was of lymphoid pathotype obtained from a subject treated with prednisone and methotrexate, but with persistently high DAS28-CRP of 4.9.

### 3D reconstructions of a Prussian blue stained PLN

A WT, Early, and Advanced PLN (n=1/group) was sectioned at 5μm through the entire lymph node. Each section was then stained with Prussian Blue and Nuclear Fast Red counterstain, then imaged using a VS120 slide scanner. Images (.tif) were exported from the slide scanner, and image stacks were imported into Amira software. The “Align Slices” module was used to rotate and align each individual section. The images were also imported into ImageJ for color segmentation using the “Color Deconvolution 2” plug-in, where blue (iron) and red (stromal cells) were segmented using the Feulgen LightGreen vector with a set threshold. The segmented red and blue sections were then exported as an image sequence, and then imported into Amira and aligned with the original images. Animations were then generated in Amira to develop the **Supplementary Videos**.

### IgG2b ELISPOT

PLNs were harvested from TNF-Tg mice with Early and Advanced arthritis (n=3 mice, 6 PLNs per group). A lethal dose of ketamine/xylazine cocktail was administered intraperitoneal prior to tissue harvest. Evan’s Blue dye (2%) was injected intradermal into the hindpaws of the mice for identification of the PLNs, then the fur on the posterior aspect of the hindlimb was removed with depilatory cream. The PLNs were incubated in 10% FBS in DMEM prior to removal of external tissue and single-cell isolation by mechanical digestion of the tissue in 3mL of 20% FBS in PBS using a scalpel. A 30μm MACS SmartStrainer (Miltenyi Biotec, Cat# 130-098-458) was pre-wet with 3 mL of 20% FBS, the cell suspensions were passed through, and 20% FBS was added until the total volume reached 10mL. Each sample was then pelleted at 300g and 4°C for 10min and resuspended in 5mL of red blood cell (RBC) lysis buffer (ThermoFisher Scientific, Cat# 00-4333-57) at room temperature. The lysis was then stopped by adding PBS up to 40mL to each sample. The cells were pelleted at 300g and 4°C, and the isolated cells were then processed following the instructions of the Mouse IgG2b Single-Color ELISPOT kit without *in vitro* stimulation (Immunospot). The wells were imaged using an ELISPOT reader (Immunospot), and spots were counted manually after importing the images into ImageJ.

### IgG isotyping serum ELISA

Blood from the Early and Advanced TNF-Tg mice used in the scRNAseq (n = 3 mice/group) was collected by cardiac puncture. The blood was allowed to sit at room temperature for 30-min and then was centrifuged at 1500x g for 10 min at 4°C. The serum was then collected, and aliquots were frozen at -80°C. Serum was then analyzed following the instructions of the Mouse IgG Isotyping kit (Abcam, Cat# ab273149).

### Statistical analysis

Linear regression, t-test, one-way ANOVA, and two-way ANOVA statistical analyses were performed using GraphPad Prism (v9.1.0, San Diego, CA, USA), as appropriate. Multiple linear regression analysis was performed in R (v4.1.2) using the “lm” function to build models with talus bone volume as the dependent variable and immunoglobulin gene expression (continuous) ± disease group (categorical, Early vs Advanced) as the independent variables. For the results of the multiple linear regression, the association with disease group on talus bone volumes is reported for the Advanced condition. Lack of multicollinearity between the independent variables was confirmed through quantification of the variance inflation factor (VIF), which was <5 for each independent variable across the models. The R^2^ values associated with the multiple linear regression models are adjusted to take into account the number of independent variables utilized in the particular model. The formula for each multiple linear regression model is provided:


Model 1=Talus~Ighg1/Ighm+Ighg2b/Ighm+Ighg2c/Ighm+Ighg3/Ighm

Model 2=Talus~Model 1+Group(Early vs Advanced)

Model 3=Talus~Ighg2b/Ighm+Ighg3/Ighm

Model 4=Talus~Model 3+Group(Early vs Advanced)

## Results

### Spatial transcriptomics identifies PLN sinus regions with enhanced immunoglobulin production in TNF-Tg mice with advanced arthritis

**Figure 1:**
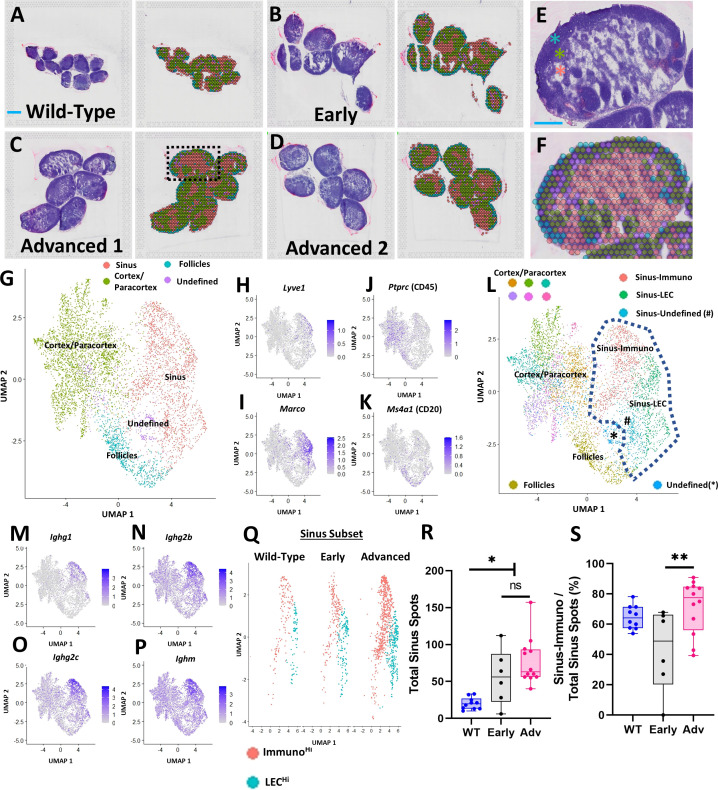
Spatial transcriptomics identifies PLN sinus regions with enhanced immunoglobulin production in TNF-Tg mice with Advanced arthritis. PLNs from WT (5.5-month-old; n=10 PLNs) **(A)** and TNF-Tg mice with Early (5-6-month-old; n=6 PLNs) **(B)** and Advanced (>8-month-old; n=12 PLNs, 6/capture area) **(C, D)** arthritis were harvested and processed for spatial transcriptomics. Each H&E-stained image (left) corresponds to a transcriptional representation (right) of the PLN sinus (red spots overlaying white sinus spaces), cortical/paracortical (green spots), and follicular (blue spots) regions. Isolated spots with high ribosomal gene content were annotated as an undefined (purple) cell population **(A–D)**. A representative high-magnification H&E image from “Advanced 1”, (dashed box) is provided to appreciate the cortex (high cell density, blue asterisk), paracortex (medium cell density, green asterisk), and sinus (low cell density, red asterisk) **(E)**, alongside the spatial gene expression and corresponding UMAP labeling the follicles (within the cortex, blue spots), cortex/paracortex (green spots), and sinuses (red spots) **(F, G)**. Feature plots represent the identification of *Lyve* ^Hi^ */Marco* ^Hi^ sinuses **(H, I)**, *Ptprc* ^Hi^ cortices/paracortices **(J)**, and *Ptprc* ^Hi^ */Ms4a1* ^Hi^ follicles **(K)** that were validated by histologic localization as in **(A–F)**. Unsupervised clustering (resolution=0.5) resolved 11 transcriptionally distinct PLN regions, where the sinus-associated spots localized to 3 distinct clusters (dashed lines) **(L)**. The *Ighg1* ^Hi^/*Ighg2b* ^Hi^/*Ighg2c* ^Hi^/*Ighm* ^Hi^ Sinus-Immuno **(M–P)** and Ig^Lo^ Sinus-LEC populations predominated, while Sinus-Undefined spots exhibited a high proportion of ribosomal genes. The predominant Sinus-Immuno (red) and Sinus-LEC (blue) populations were subset **(Q)**, and individual PLNs were evaluated for total sinus-associated spots (Sinus-Immuno + Sinus-LEC) **(R)** and proportion of Sinus-Immuno spots **(S)**. Statistics: One-way ANOVA, *p<0.05, **p<0.01, # = Sinus-Undefined population; ns = non-significant **(R, S)**. Blue scale bar = 1mm **(A–D)** & 0.5mm **(E, F)**.

To gain deeper insights into the dynamic cellular changes in the PLN sinuses of TNF-Tg mice (13), we turned to a spatial transcriptomics approach that details gene expression alterations during disease progression in histologically defined sinus regions. For visualization of spatial gene expression, representative H&E-stained images of the PLN tissues and clustering of spatial transcriptomics datasets from wild-type and TNF-Tg mice with Early and Advanced arthritis are presented in **Figures 1A–D**. To highlight the distinct PLN microstructure and the corresponding spatial gene expression, a high magnification image is provided (**Figures 1E, F**; derived from black dashed box in **Figure 1C**). The lymph node contains 3 distinct general anatomic regions: cortex (blue asterisk, high cell density), paracortex (green asterisk, medium cell density), and sinuses (red asterisk, low cell density) (**Figure 1E**). Note the remarkable specificity of the red spatial spots to the regions of the PLN with low cell density, denoting a particular sinus-related gene expression pattern (**Figure 1F**). The sinuses connect the multiple afferent lymphatic vessels draining into the lymph node culminating in an efferent lymphatic vessel at the medulla that traffics lymph away from the lymph node. Circulating immune cells also enter the lymph node via high endothelial venules in the paracortex. Lymph node follicles exist within the cortex where B-cells typically accumulate through a CXCL13 gradient produced by follicular dendritic cells (33–35). When activated, B-cells migrate into the cortical/paracortical peri-follicular regions to interact with T-cells and monocyte-derived cells (macrophages, dendritic cells) relaying cytokine- and/or antigen-associated signals (36, 37).

A UMAP plot of the integrated datasets with minimally supervised clustering (manual combination of initial clusters based on anatomical localization) corresponding with the anatomical regions of the PLN noted in **Figures 1E, F** (green spots, cortex/paracortex; blue spots, follicles; and red spots, sinuses) is provided to further visualize gene expression patterns (**Figure 1G**). Given the cell heterogeneity within the cortex/paracortex, and the relatively low-resolution sequencing of the spatial spots, we opted to maintain the cortex/paracortex together as a single population to generate a comparison between the sinuses and surrounding lymph node parenchyma as a whole. The follicles were separated given the specific cellular localization of B-cells within these regions, further supported by differential gene expression from the remainder of the cortex/paracortex. Specifically, the spatially localized gene expression analysis identified the PLN sinuses as *Lyve1* ^Hi^/*Marco* ^Hi^ (**Figures 1H, I**), consistent with markers of lymphatic endothelial cells (LECs) described in prior transcriptomic studies (24). The cell-dense regions of the cortex/paracortex were populated by hematopoietic cells based on high expression of *Ptprc* (CD45)^Hi^ (**Figure 1J**), and the B-cell follicles were enriched with *Ptprc* ^Hi^/*Ms4a1* (CD20)^Hi^ expression (**Figure 1K**). Further unsupervised clustering (resolution = 0.5) identified 11 unique spot clusters where the sinus regions of the PLN (within blue-dashed line) contained two predominant clusters: Sinus-Immuno (Ig^Hi^) and Sinus-LEC (Ig^Lo^) (**Figure 1L**). The Sinus-Immuno cluster demonstrated enrichment of immunoglobulin gene expression, such as *Ighg1*, *Ighg2b*, *Ighg2c*, and *Ighm* (**Figures 1M–P**). These predominant sinus clusters were then subset for each condition (**Figure 1Q**) to quantify and compare the spot abundance of Sinus-Immuno vs Sinus-LEC. PLNs from both TNF-Tg mice with Early and Advanced arthritis exhibited an increased number of total sinus spatial spots compared to WT (**Figure 1R**; WT 20.0 ± 8.0, Early 56.2 ± 37.6, Advanced 77.3 ± 32.0 total sinus spots), while Advanced PLNs showed a greater proportion of Sinus-Immuno spots compared to Early PLNs (**Figure 1S**; WT 64.6 ± 7.8, Early 43.1 ± 27.0, Advanced 71.6 ± 17.7% Sinus-Immuno spots).

### PLN expansion of *Marco* ^+^ peri-follicular medullary sinuses determined by spatial and single-cell integration

**Figure 2:**
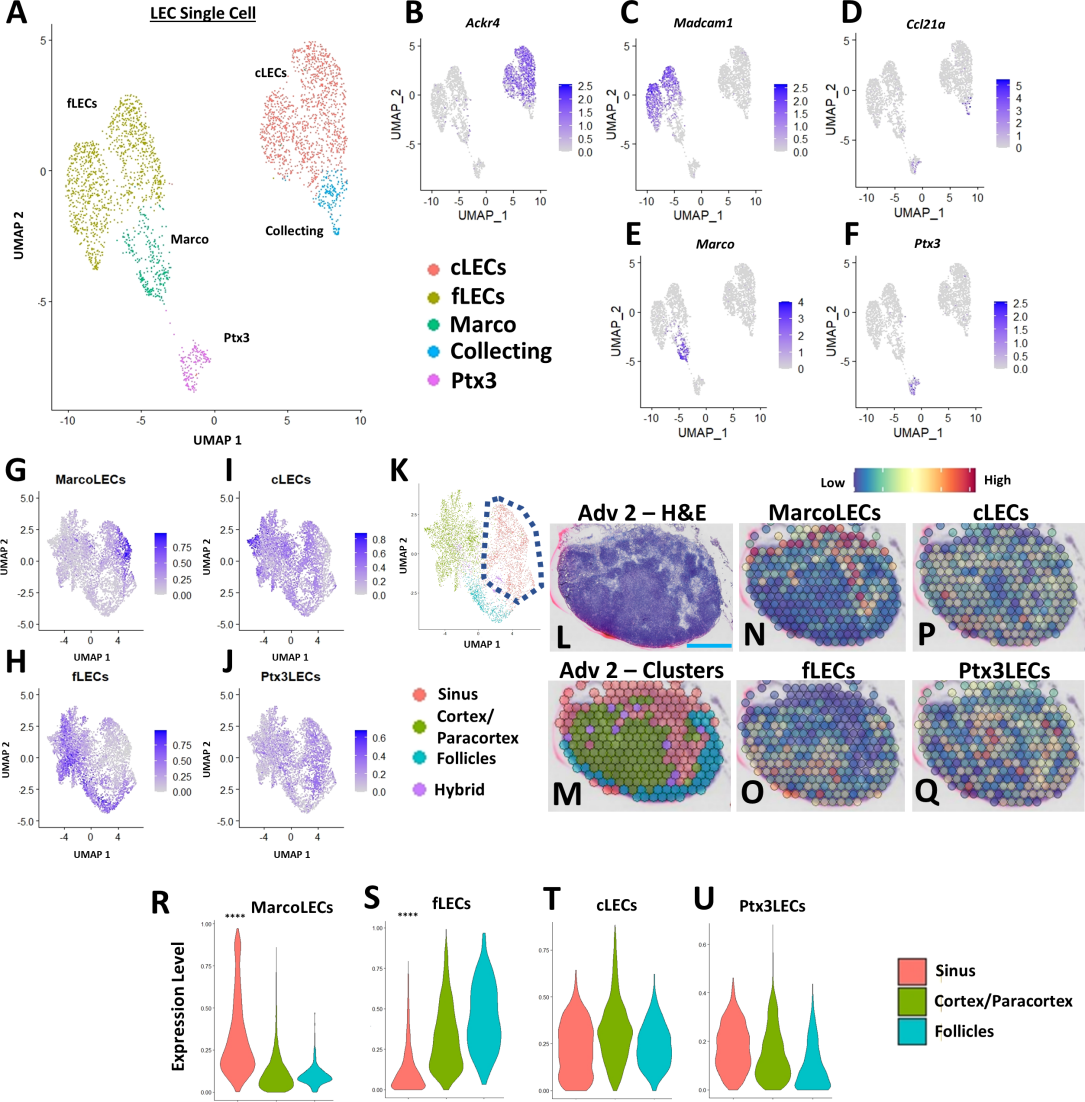
PLN expansion of *Marco* ^+^ peri-follicular medullary sinuses determined by spatial and single-cell integration. Subpopulations of LECs from a published scRNAseq dataset of C57BL/6 murine lymph nodes [ (24), GSE145121] were visualized in a UMAP **(A)**. Specific LEC populations were defined by gene enrichment **(B–F)**, as previously described (24). The LEC single-cell data was integrated with the regions identified by spatial transcriptomics, where feature plots demonstrate the selective enrichment for Marco-LECs in the sinuses **(G)**, fLECs in the cortices/paracortices and follicles **(H)**, and the general distribution of cLECs **(I)** and Ptx3-LECs **(J)** throughout the spatially-resolved PLN regions **(K)**, identified in **Figure 1**. A representative H&E-stained image of the Adv 2 capture area **(L)** with cluster annotation **(M)** is shown (high-magnification from **Figure 1D**) as a spatial feature plot to further demonstrate the Marco-LECs localized to the expanded sinus regions of TNF-Tg PLNs **(N–Q)**. Quantification of Marco-LECs **(R)**, fLECs **(S)**, cLECs **(T)**, and Ptx3-LECs **(U)** within the spatially-resolved PLN regions confirmed a significant enrichment for Marco-LECs (Log2FC = 0.354) within and fLECs (Log2FC = -0.321) outside of the PLN sinuses. Statistics: Wilcoxon Rank-Sum Test of sinus vs. all other regions; ****FDR< 2.23E-308 **(R, S)**. Blue scale bar = 0.5mm **(L–Q)**.

Due to the dynamic cellular changes within the TNF-Tg PLN sinuses during the onset and progression of inflammatory-erosive arthritis, we aimed to elucidate the identity of the affected sinus regions. To localize the expanded TNF-Tg PLN sinus regions with distinct LEC populations, we integrated our spatial transcriptomics with a published scRNAseq dataset of lymph node LEC subtypes (24) (**Figure 2A**), including those localized to the *Ackr4* ^+^ subcapsular ceiling (cLECs), *Madcam1* ^+^ subcapsular floor (fLECs), *Ccl21a^+^* collecting vessel, *Marco^+^* peri-follicular medullary, and *Ptx3^+^* central medullary sinuses (**Figures 2B–F**). We then computed phenotype prediction scores for each spatial spot corresponding to the LEC subpopulations generated from the scRNAseq dataset by using *FindTransferAnchors* function in Seurat and visualized each LEC subtype on the UMAP defined by the spatial transcriptomics analysis (**Figures 2G–J**). The Marco-LECs were associated with the expanded sinus regions in TNF-Tg mice (**Figure 2K**, red cluster within dashed lines with notable enrichment of Marco-LECs shown in **Figure 2G**). This spatial identity of increased *Marco* ^+^ sinuses in TNF-Tg lymph nodes was confirmed by histology and spatial transcriptomics of sinus regions (**Figures 2L, M**, red spots; representative PLN reproduced from **Figure 1D** for convenience) that showed selective spatial localization of Marco-LECs (**Figure 2N**, red = high, blue = low expression) relative to other LEC subtypes (**Figures 2O–Q**) in the PLN. Quantitative analysis confirmed a significant enrichment for Marco-LEC related gene expression within the spatially-defined sinus regions (**Figure 2R**) compared to cortex/paracortex and follicle regions (**Figures 2S–U**). Thus, by integrating scRNAseq and spatial transcriptomics datasets, we identified the *Marco* ^+^ peri-follicular medullary sinuses as those involved in PLN “Expansion” and “Collapse” during TNF-Tg arthritis progression.

### Aggregation of IgG2b^+^ plasma cells adjacent to PLN MARCO^+^ sinuses is associated with exacerbation of erosive arthritis in advanced TNF-Tg mice

**Figure 3:**
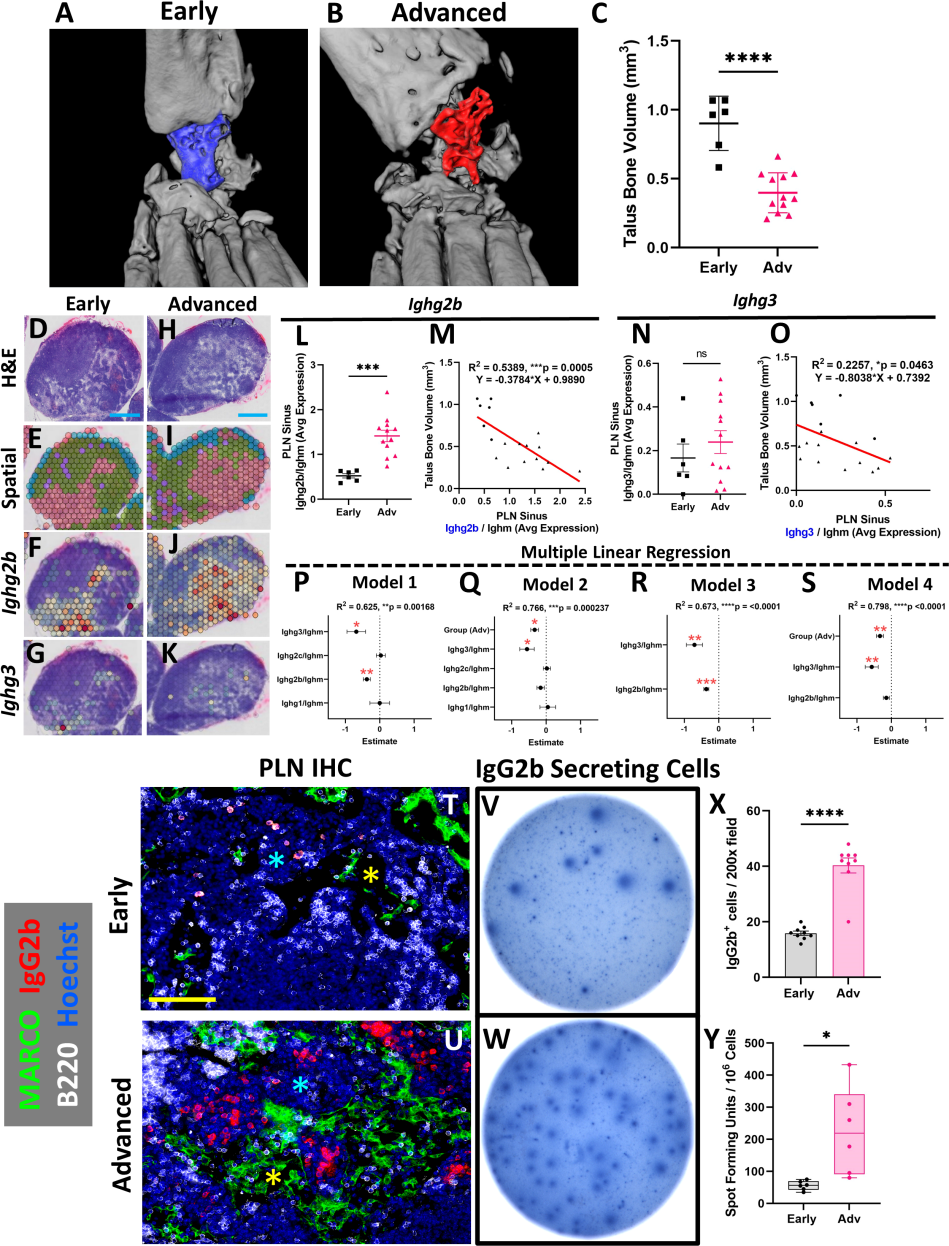
Aggregation of IgG2b^+^ plasma cells adjacent to PLN MARCO^+^ sinuses is associated with exacerbation of erosive arthritis in advanced TNF-Tg mice. *Ex-vivo* micro-CT of TNF-Tg hindpaws with Early **(A)** and Advanced **(B)** arthritis showed significantly reduced talus bone volumes in Advanced joints **(C)**. A transcriptional representation of the sinus regions (red; green = cortex/paracortex, blue = follicles, purple = undefined) and spatial feature plots of *Ighg2b* or *Ighg3* expression (red = high, blue = low expression) for Early **(D–G)** and Advanced (i.e. Adv 2) **(H–K)** PLNs are shown (**D, E, H, I** are high-magnification from **Figures 1B, D** (rotated)). There was a significant increase in *Ighg2b/Ighm* expression ratio in Advanced vs Early PLNs **(L)**, along with a significant and negative correlation of *Ighg2b/Ighm* sinus expression with talus bone volumes (R^2 ^= 0.54, *p<0.001*; circles = Early, triangles = Adv) **(M)**. While *Ighg3/Ighm* was unchanged between Early vs Adv **(N)**, there was also a significant negative correlation with talus bone volumes (R^2 ^= 0.23, *p=0.046*) **(O)**. Forest plots representing multiple linear regression revealed that *Ighg2b/Ighm* and *Ighg3/Ighm* vs talus correlation was independent of other immunoglobulins **(P)**, while the effect of *Ighg2b/Ighm* is dependent on disease stage **(Q)**. Combination of *Ighg2b/Ighm* and *Ighg3/Ighm* vs talus improved the correlation (**R**, R^2^ = 0.67, *p<0.0001*), although *Ighg2b/Ighm* was similarly associated with onset of Advanced arthritis **(S)**. The increase in IgG2b^+^ cells in Advanced TNF-Tg PLNs was validated by immunofluorescence where IgG2b^+^ cells were limited in Early PLNs **(T)**, while Advanced PLNs **(U)** exhibited a significant accumulation of IgG2b^+^/B220^-^ (red/white) plasma cells **(X)** in the medullary cords (blue asterisks in **T, U**) localized to the MARCO^+^ (green) PLN sinuses (yellow asterisks in **T, U**). IgG2b ELISPOT **(V, W)** further confirmed significantly increased IgG2b secreting cells in Advanced PLNs **(Y)**. Statistics: Unpaired t-test **(C, L, N, X, Y)**; linear regression **(M, O)**; multiple linear regression **(P–S)**; *p<0.05, **p<0.01, ***p<0.001, ****p<0.0001. Blue scale bar = 0.5mm **(D–K)**, yellow scale bar = 100μm **(T, U)**.

To examine gene expression changes in the *Marco* ^+^ sinus regions of the TNF-Tg PLNs and correlation with the severity of inflammatory-erosive arthritis in the afferent ankle joint, *ex-vivo* micro-CT of the hindpaws was performed (**Figures 3A, B**; note the dislocation of talus bone from tibia in Advanced arthritis). As expected, the mice with Advanced arthritis had significantly decreased talus bone volumes compared to mice with Early arthritis (**Figure 3C**; Advanced 0.40 ± 0.15 vs Early 0.90 ± 0.20mm^3^, *p<0.0001*). To investigate the relationship between sinus-related gene expression and talus bone volumes in the afferent ankle joint, we evaluated the average gene counts within the red sinus spatial spots, exemplified by *Ighg2b* and *Ighg3* expression, in representative Early (**Figures 3D–G**) and Advanced (**Figures 3H–K**) PLNs (reproduced from **Figures 1B, D** [rotated], for convenience). Identified first through differential gene expression in Advanced vs Early sinuses, *Ighg2b* (log_2_ fold-change: 1.04, *FDR=1.12E-31* for Advanced vs Early) was evaluated as an expression ratio with *Ighm* to correct for the estimated B-cell abundance across PLNs (described in Materials and Methods, Spatial Transcriptomic Analysis). We observed that TNF-Tg mice with Advanced arthritis showed significantly greater *Ighg2b*/*Ighm* expression compared to those with Early arthritis (**Figures 3L**; Early 0.52 ± 0.12 vs Advanced 1.41 ± 0.45 counts/counts, *p<0.001*). Importantly, expression levels of *Ighg2b/Ighm* demonstrated a significant negative correlation with talus bone volumes (**Figure 3M**; R^2^ = 0.54, *p=0.0005*), suggesting a strong relationship between arthritic severity and immune responses in the joint-draining PLNs.

We next investigated the complete panel of IgG immunoglobulin genes between Early and Advanced TNF-Tg mice, along with the relationship with talus bone volumes. Our findings demonstrated that *Ighg1, Ighg2a*, *Ighg2c*, and *Ighg4* either showed no association with joint disease or were undetectable in the spatial dataset (**Supplementary Figure 3**, IgG immunoglobulin genes were excluded if undetectable). On the other hand, while *Ighg3* showed no difference between Early and Advanced stages of arthritis (**Figure 3N**; Early 0.17 ± 0.16 vs Advanced 0.24 ± 0.18 counts/counts, *p>0.05*), *Ighg3* exhibited a significant, albeit weak, correlation with talus bone volumes (**Figure 3O**; R^2^ = 0.23, *p=0.046*). Given both *Ighg2b/Ighm* and *Ighg3/Ighm* showed significant negative correlations with talus bone volume, we performed a multiple linear regression to control for the effects of the differentially expressed immunoglobulins and the stage of arthritis (Early vs Advanced). Our investigation demonstrated that the specific immunoglobulin and bone volume relationships were independent of the other immunoglobulins (**Figure 3P**; Model 1 Estimates: *Ighg2b/Ighm* -0.377 ± 0.102 & *Ighg3/Ighm* -0.683 ± 0.27, *p<0.05*). However, the correlation of *Ighg2b/Ighm* and talus bone volumes was dependent on Early vs Advanced disease (**Figure 3Q**; Model 2 Estimates: *Ighg2b/Ighm* -0.161 ± 0.108, *p>0.05* & *Ighg3/Ighm* -0.556 ± 0.21, *p<0.05*). Interestingly, the *Ighg3/Ighm* association with talus bone volumes remained consistent regardless of Early vs Advanced arthritis. Based on these findings, we evaluated the combined effect of *Ighg2b/Ighm* and *Ighg3/Ighm* levels on talus bone volumes, which together showed improved correlation (**Figure 3R**; Model 3: R^2^ = 0.67, *p<0.0001*). Addition of Early vs Advanced disease further enhanced the relationship with talus bone volumes, but similarly negated the dependent *Ighg2b/Ighm* effect (**Figure 3S**; Model 4: R^2^ = 0.80, *p<0.0001*). Together, these findings suggest that relative levels of *Ighg3/Ighm* may be related to a basal rate of inflammatory-erosive activity explaining differences in arthritis at the same stage of disease. On the other hand, late-onset *Ighg2b/Ighm* activity corresponds with exacerbation of arthritic flare and lymph node collapse associated with Early vs Advanced stages of disease, which was investigated further.

Additional detailed evaluation of *Ighg2b* through immunofluorescence showed a limited accumulation of IgG2b^+^ cells in Early TNF-Tg PLNs (**Figure 3T**), while IgG2b^+^/B220^-^ cells localized to the medullary cords directly adjacent to the MARCO^+^ sinus tissue and were significantly increased in Advanced PLNs (**Figures 3U, X**; Early 15.9 ± 2.3 vs Advanced 40.3 ± 8.1 IgG2b^+^ cells/200x field, *p<0.0001*). Advanced PLNs also exhibited an increase in IgG2b secreting cells by ELISPOT, which together confirm the results of the spatial transcriptomics analysis (**Figures 3V, W, Y**; Early 55.8 ± 14.6 vs Advanced 225.8 ± 135.4 spot-forming units per million cells, *p<0.05*). Furthermore, IgM^+^ cells were found to be significantly reduced in Advanced PLNs, associated with the increase in IgG^+^ cells and without signs of a corresponding change in cell proliferation by PCNA staining, supporting the identity of non-cycling plasma cells (**Supplementary Figure 4**). Remarkably, despite an increase in plasma and IgG2b-antibody secreting cells, there was no difference in IgG2b antibody levels in the serum of Advanced mice measured by ELISA (**Supplementary Figure 6G**). The IgG2b isotype was also the predominant immunoglobulin in both Early and Advanced mice, as previously reported in C57BL/6 mice (38). Together, these data suggest that plasma cell differentiation and IgG2b class-switching in joint-draining lymph nodes are integral to the onset of Advanced arthritis in TNF-Tg mice.

### Accumulation of synovial and PLN sinus iron-laden macrophages are associated with advanced arthritis

**Figure 4:**
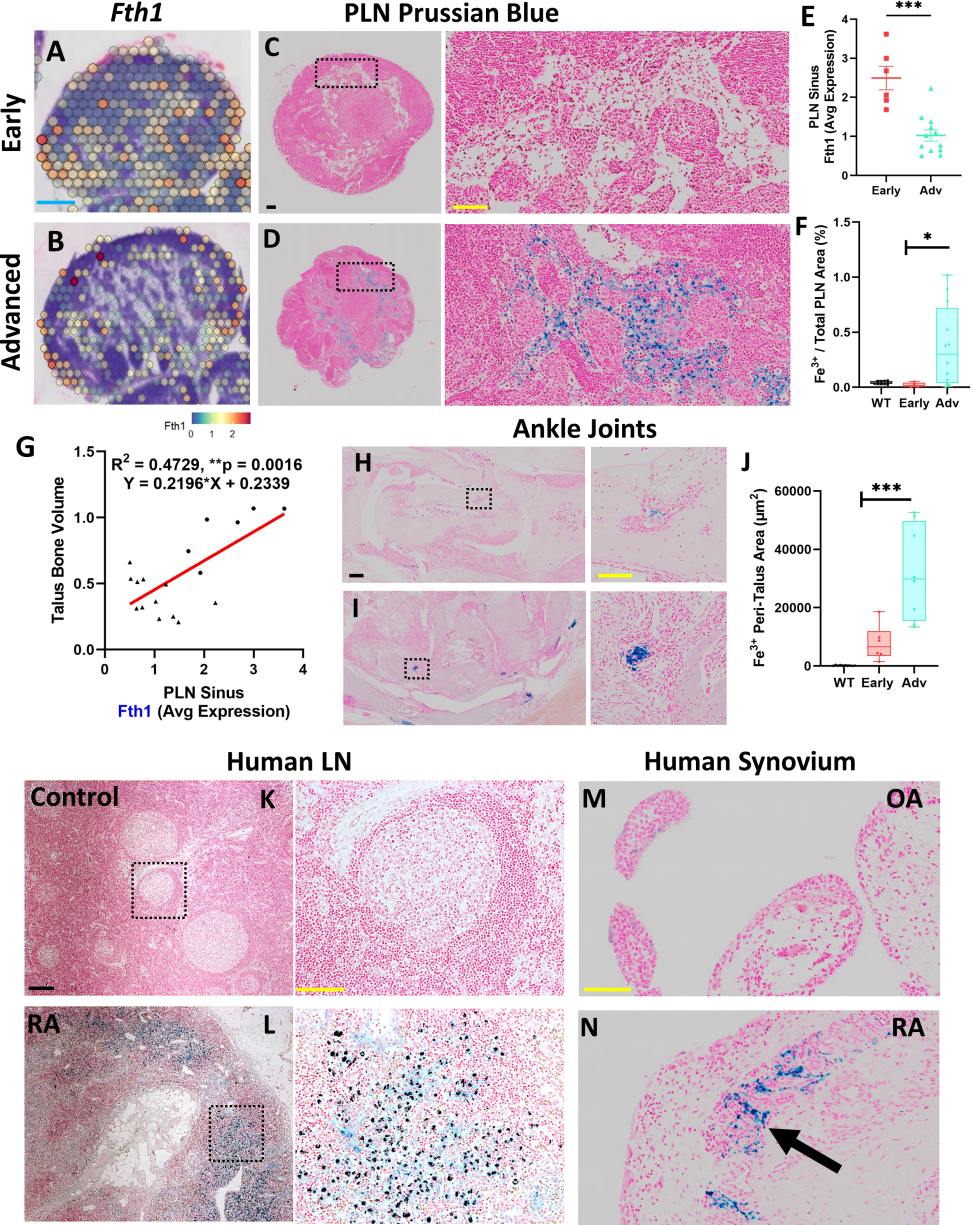
Accumulation of synovial and PLN sinus iron-laden macrophages are associated with advanced arthritis. Differential gene expression analysis within the PLN low cell density sinus regions revealed reduced expression of *Fth1* (ferritin heavy chain 1) in Advanced compared to Early PLNs (red = high, blue = low expression; feature plots of high-magnification images from **Figures 1B, C**) associated with increased Prussian blue positive iron-laden cells in Advanced PLNs **(A–F)**. Of note, sinus *Fth1* levels were positively correlated with talus bone volumes in the ipsilateral ankles (R^2^ = 0.47, *p<0.01*; circles = Early, triangles = Adv) **(G)**. Similarly, the ankle synovium showed a significantly increased abundance of iron-laden cells in Advanced versus Early TNF-Tg joints **(H–J)**, suggesting that PLN-localized iron-laden cells may derive in part from the afferent synovial tissue. Towards clinical translation, human lymph node samples similarly exhibited a remarkable accumulation of Prussian blue positive cells within the lymph node sinuses of RA subjects compared to controls **(K, L)**. Synovial samples from subjects with OA compared to RA also demonstrated the clinical correlate of the iron-laden cell phenotype with Prussian blue positive cells (black arrow) detected in RA synovial tissue **(M, N)**. Statistics: Unpaired t-test **(E)**, linear regression **(G)**, and one-way ANOVA with Tukey’s multiple comparisons **(F, J)**; *p<0.05, **p<0.01, ***p<0.001. Blue scale bar = 0.5mm **(A, B)**, yellow scale bar = 100μm, black scale bar = 200μm **(C, D, H, I, K–N)**.

To further elucidate the cellular dynamics within the TNF-Tg PLN sinus regions associated with the lymphocyte activation and plasma cell accumulation, we performed differential gene expression analysis between Early and Advanced cohorts. Specifically, ferritin heavy chain (*Fth1*) that encodes the heavy subunit of ferritin (the major iron storage protein) was significantly reduced in Advanced PLN sinuses relative to Early PLNs (Early 2.5 ± 0.74 vs Advanced 1.0 ± 0.50 counts, *p<0.001*). These findings corresponded with a remarkable accumulation of Prussian blue positive iron-laden macrophages localized in Advanced PLN sinuses (**Figures 4A–F**, WT 0.043 ± 0.014% vs Early 0.020 ± 0.021% vs Advanced 0.37 ± 0.36% Prussian blue to total PLN area, *p<0.05*). We also performed 3D reconstructions of a Prussian blue stained PLN from each group and confirmed the global accumulation of iron-laden cells selective for Advanced PLNs (**Supplementary Videos**). Of note, the dichotomy of reduced *Fth1* gene expression associated with increased iron-laden macrophages suggests detection of a negative feedback response to intracellular iron accumulation during RA progression. Additionally, *Fth1* expression levels were positively correlated with talus bone volumes in ipsilateral ankles (**Figure 4G**, R^2^ = 0.47, *p<0.01*). As macrophages are critical for regulating iron homeostasis (39), we further confirmed the identity of the Prussian blue positive cells as iron-laden macrophages by their immediate proximity to F4/80^+^/MARCO^+^ macrophages aggregated within PNAd^-^/LYVE1^+^/MARCO^+^ LEC lined PLN sinuses (**Supplementary Figure 5**). To evaluate the potential origins of these iron-rich macrophages, we next assessed ankle joints by Prussian blue staining, which showed a corresponding dramatic increase in iron-laden macrophages in the synovium of TNF-Tg mice with Advanced arthritis (**Figures 4H–J**; WT 163.3 ± 117.7 vs Early 7850 ± 6090 vs Advanced 31897 ± 16007 μm^2^ of Prussian blue in the peri-talus region; *p<0.001*).

We then performed a preliminary clinical correlate study of the iron-laden macrophage phenotype with an assessment of Prussian blue staining in human lymph nodes and synovia. Remarkably, lymph nodes from RA patients similarly demonstrated an accumulation of Prussian blue positive cells within the sinus region relative to controls without known history of RA (**Figures 4K, L**). In addition, relative to osteoarthritic synovium with a paucity of Prussian blue positive cells (**Figure 4M**), iron-laden macrophages were abundant within RA synovium during active disease (**Figure 4N**, black arrow). Given the limited number of iron-laden macrophages in Early arthritis in TNF-Tg mice, we conclude that inflammation alone is insufficient for the accumulation of iron in macrophages, and hypothesize that additional inciting events (e.g., phagocytosis of dead and dying cells and ferroptosis) are necessary to promote their generation and aggregation, which may be relevant to the clinical pathogenesis of RA.

### Co-stimulation of ALCAM^+^ macrophages and CD6^+^ T-cells potentially promote IgG2b^+^ plasma cell differentiation in advanced TNF-Tg PLNs

**Figure 5:**
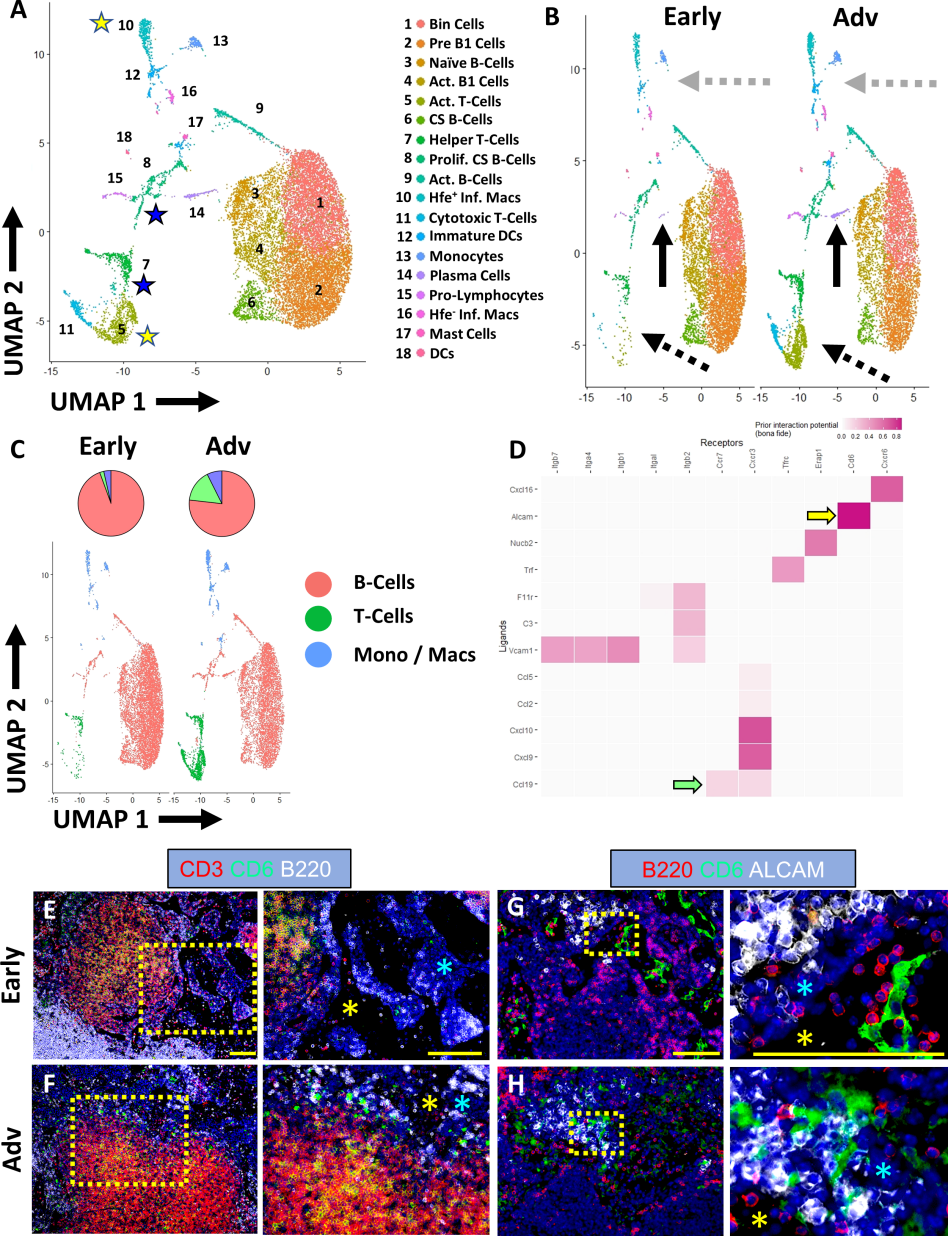
Co-stimulation of ALCAM^+^ macrophages and CD6^+^ T-cells potentially promote IgG2b^+^ plasma cell differentiation in advanced TNF-Tg PLNs. Single-cell RNA-sequencing resolved 18 distinct cell clusters **(A)**, representing subtypes of B-cells, T-cells, and monocytes/macrophages (annotations in **Supplementary Table 2**). Increased plasma cells (solid black arrows), T-cells (dashed black arrows), and macrophages (dashed grey arrows) were identified in Advanced PLNs **(B, C)**. Cell-cell interaction analysis between *Hfe^+^/Cx3cr1^+^/Cd88^+^/Aif1^+^* inflammatory macrophages (ligands) and *Ccr7^+^/Cd27^+^/Cd96^+^/Cd226^+^* activated T-cells (receptors) (yellow stars in **A**) revealed high interaction potential between *Alcam^+^* macrophages and *Cd6^+^* T-cells (yellow arrow) with potential *Ccr7*/*Ccl19* (green arrow) recruitment mechanisms **(D)**. Immunostaining confirmed the presence of CD3^+^/CD6^+^ T-cells **(E, F)** with the physical separation of ALCAM^+^ and CD6^+^ cells in Early PLNs **(G)** and close proximity in Advanced arthritis **(H)**, which occurs in the medullary cords (blue asterisks) directly adjacent to the PLN sinuses (**E–H**, yellow asterisks). Yellow scale bar = 100μm **(E–H)**.

Based on the findings of increased IgG2b^+^ plasma cells and iron-laden macrophages localized to the MARCO^+^ sinus region in Advanced PLNs, we evaluated changes in these cell populations by scRNAseq during Early and Advanced arthritis. Following integration of the Early (8192 cells) and Advanced (8136 cells) datasets, unsupervised clustering (resolution = 0.5) resolved 18 unique cell populations (complete annotations provided in **Supplementary Table 2**). As expected, scRNAseq revealed a predominance of Bin cells in TNF-Tg PLNs (**Figure 5A**). Interestingly, comparison of the cellular composition of Early versus Advanced PLNs revealed a striking increase in the number of T-cells (**Figure 5B**, dashed black arrows; **Figure 5C**, 2.0% Early vs 15.8% Advanced of clusters 5, 7, and 11 combined relative to total cells). Similarly, the monocyte/macrophage populations exhibited a notable increase in the Advanced condition (**Figure 5B**, dashed grey arrows; 3.6% Early vs 7.4% Advanced of clusters 10, 12, 13, 16, and 18 combined relative to total cells). These proportional changes between the conditions are highlighted in **Figure 5C**; **Supplementary Table 2**.

The scRNAseq also further validated the spatial transcriptomics and immunofluorescent analysis with a remarkable increase in the proportion of *Cd93^+^/Irf4^+^*/*Cxcr4^+^* plasma cells in Advanced PLNs (**Figure 5B**, solid black arrows; 0.08% Early vs 2.0% Advanced relative to total B-cells). Through investigation of immunoglobulin expression within the B-cell populations, we demonstrated the ubiquitous expression of *Ighm* within all B-cells (**Supplementary Figure 6A**). In addition, we confirmed the increase in *Ighg2b* levels in Advanced compared to Early TNF-Tg PLNs. However, we unexpectedly noted that without spatial differentiation, single-cell *Ighg2b* gene expression was elevated in all B-cell subsets and was not exclusive to differentiated plasma cells (**Supplementary Figure 6B**). Of note, the increase in *Ighg2b* expression in B-cells throughout the PLN was also much higher relative to other immunoglobulins, where *Ighg1*, *Ighg2a/c*, *Ighg3*, and *Ighg4* showed limited or absent levels in both the Early and Advanced datasets (**Supplementary Figures 6C–F**).

Given the robust validation of IgG2b^+^ class-switching with increased propensity towards plasma cell phenotype in the B-cells in Advanced PLNs (**Figure 3**), we next evaluated the potential for macrophage/T-cell stimulation leading to the differentiation of B-cells towards IgG2b class-switched plasma cells. We performed cell-cell interaction analysis of the clusters in the single-cell datasets using NicheNet R package (23). Analysis of *Hfe^+^*/*Cx3cr1^+^*/*Cd88^+^*/*Aif1^+^* inflammatory macrophages and *Ccr7^+^*/*Cd27^+^*/*Cd96^+^*/*Cd226^+^* activated T -cells (yellow stars in **Figure 5A** and yellow rows in **Supplementary Table 2**) showed a strong interaction potential between *Alcam* (macrophages) and *Cd6* (T-cells) (**Figure 5D**, yellow arrow). Of note, ALCAM/CD6 interaction is a co-stimulatory pathway involved in T-cell activation (40, 41), and anti-CD6 clinical trials for RA are currently underway with promising outcomes (42, 43). Chemotactic signals, such as recruitment of *Ccr7* ^+^ T-cells via *Ccl19* produced by macrophages, were also identified as a putative explanation for the dramatic increase in T-cells in Advanced arthritis (**Figure 5D**, green arrow). However, immunofluorescent evaluation primarily demonstrated CCL19 expression within stromal tissue adjacent to the F4/80^+^ and/or MARCO^+^ macrophages accumulated around the Advanced PLN sinuses (**Supplementary Figure 7**). The activated *Cd4^+^*/*Cd8^-^*/*Cd40lg^+^* helper T-cells were also analyzed against the class-switching and proliferating *Nme1/2^+^*/*Mki67^+^*/*Top2a^+^*/*Jchain^+^* B-cells (blue stars in **Figure 5A** and blue rows in **Supplementary Table 2**). The interaction analysis identified well-known pathways including CD40LG (T-cell) and CD40 (B-cell) co-stimulation required for B-cell activation (44, 45), and IL21 (T-cell) and IL21R (B-cell) promoting plasma cell differentiation (**Supplementary Figure 6H**, blue arrows) (46). Immunofluorescence of Early and Advanced PLNs confirmed the presence of CD3^+^/CD6^+^ T-cells adjacent to PLN sinuses (**Figures 5E, F**). Notably, while Early PLNs demonstrated a physical separation between CD6/ALCAM expression (**Figure 5G**), CD6^+^ T-cells and ALCAM^+^ macrophages exhibited remarkably close proximity within the medullary cords adjacent to the sinuses in the Advanced condition (**Figure 5H**), suggesting increased cell-cell interaction potential as predicted by the bioinformatic analysis. Taken together, the findings of increased IgG2b^+^ plasma cells in TNF-Tg Advanced PLNs with ALCAM/CD6 co-stimulatory signaling support our hypothesis that these events may be the nidus for immune mediated flare during chronic inflammatory arthritis.

## Discussion

B-cell translocation into sinuses is known to be integral to the pathogenesis of PLN “Collapse” and initiation of Advanced inflammatory-erosive arthritis in TNF-Tg mice (4), but identification of distinct mechanisms involved in this process have been limited without adequate spatial resolution and analysis. Using multi-omic spatial and scRNAseq techniques, we identified dynamic changes in the cell populations and their transcriptomic profiles localized to MARCO^+^ peri-follicular medullary sinuses of TNF-Tg PLNs during the progression of arthritis development. These alterations may be responsible for chronic inflammation induced immune activation and lymphatic dysfunction in RA. Previously, the transition from PLN expansion (Early arthritis) to PLN collapse (Advanced arthritis) was predominantly attributed to mechanical inhibition of fluid flow through B-cell accumulation in the sinuses (4).

Herein, we demonstrate a significant increase in IgG2b class-switching and accumulation of plasma cells in Advanced vs Early PLN sinuses, which corresponds to the enigmatic process of lymph node collapse and severe exacerbation of arthritic flare in late-stages of TNF-Tg joint disease (13). Further, we identified a novel relationship between *Ighg3* expression and severity of erosive arthritis independent of disease stage, which may relate to the variation of arthritis progression within TNF-Tg cohorts at the same age. Although the particular functions of IgG subtypes is primarily derived from understanding in humans, IgG2 antibodies are typically considered as a response to bacterial pathogens, which is interesting given the long-prevailing concept that RA pathogenesis is triggered by immunologic response to microbiota (47). Given the relationship between IgG2b and arthritic severity, along with the known response of IgG2 to infectious pathogens, a detailed investigation into the microbiome of TNF-Tg mice is warranted. On the other hand, IgG3 is a pro-inflammatory immunoglobulin with a short half-life, thought potentially to function towards dampening overactive inflammatory responses (48). Interestingly, a very recent study identified that human synovial lymphocytes exhibit enhanced class-switching to *IGHG3* and *IGHG1* (the most common IgG isotype in humans (48), similar in abundance to *Ighg2b* in mice) with corresponding systemic manifestations associated with the degree of synovial lymphocytic infiltration in RA (49). Our findings of *Ighg2b* and *Ighg3* expression in joint-draining PLNs related to arthritic progression provide interesting similarities and further expand upon this novel study. However, the exact mechanistic relationship between IgG3 and arthritic severity remains unclear, where IgG3 may simply serve as a surrogate measure of differences in systemic inflammation between mice, or IgG3 could be directly involved in the mediating pathologic bone erosions. Given the novel findings of *Ighg3* expression directly related to arthritis regardless of age, a detailed evaluation of the potential mechanisms of *Ighg3*-mediated erosive activity is warranted, which may further elucidate the differences in the rate of arthritic progression in otherwise age-matched TNF-Tg mice.

Overall, these findings provide support for antigen-specific reactions and adaptive immune responses, never before appreciated in the TNF-Tg mouse model of RA (12, 50). These findings also provide novel insights into previously reported B-cell effector functions in TNF-Tg mice, such as Bin cell translocation into PLN sinuses and identification of B-cells in the synovium and subchondral bone that promote erosion (51). Despite the robust IgG2b class-switching in the PLNs, the lack of changes in serum IgG2b level was unexpected. Future experiments are warranted to evaluate the antigen-specificity and B-cell receptor diversity associated with the IgG2b class-switching and the clonality of this humoral response. In addition, elucidating the mechanisms involved in the production of IgG2b^+^ cells are also required given the significant correlation of PLN sinus *Ighg2b* expression with the severity of erosive arthritis in afferent ankles and association with the progression from Early to Advanced arthritic flare.

Moreover, the cellular dynamics in joint-draining lymph nodes in Advanced arthritis are far from limited to B-cells, but also exhibit substantial increases in macrophage and T-cell populations that likely regulate plasma cell differentiation. Through our spatial transcriptomics analysis, we identified down-regulation of Ferritin (*Fth1*) gene expression within the PLN sinuses of TNF-Tg mice with Advanced arthritis, which was associated with accumulation of iron-laden macrophages. Our results also suggest strong cell-cell interaction between ALCAM^+^ inflammatory macrophages and CD6^+^ recruited T-cells. Importantly, the ALCAM/CD6 co-stimulatory pathway has been implicated in collagen-induced arthritis and other models of autoimmunity (52, 53), and phase 1 clinical trials with anti-CD6 monoclonal antibody therapy in RA patients produced encouraging results (42, 43). However, our current study is limited by lack of evidence for *bona fide* ALCAM/CD6 interaction, and investigation on the specific downstream effects of these cellular mechanisms, such as IgG2b class-switching of adjacent B-cells. Thus, targeting this pathway to evaluate the effects on TNF-Tg arthritis and lymphatic pathology is warranted. Specifically, future studies ought to be evaluated through targeted anti-CD6 inhibitors in TNF-Tg mice (54), or through genetic ablation of CD6 by crossing CD6-KO with TNF-Tg cohorts (55), to investigate endpoints such as arthritis severity and IgG2b^+^ plasma cell accumulation in downstream PLNs. Such investigation will be critical for evaluating the proposed role for ALCAM/CD6 co-stimulation, and further substantiate the therapeutic potential of CD6 blockade in RA.

**Figure 6:**
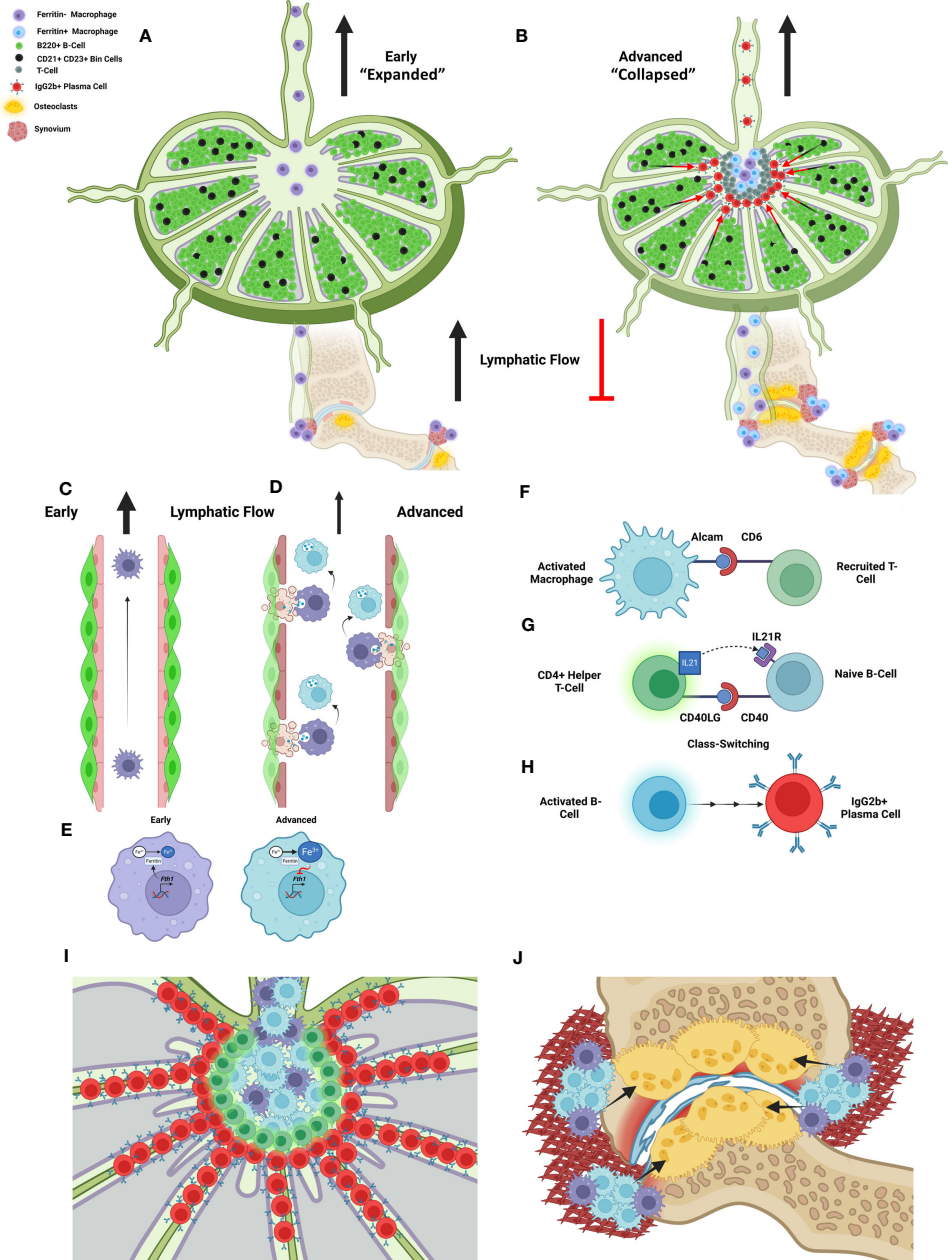
A new model of lymphatic failure and lymph node collapse with seronegative plasmocytic arthritic flare. We provided spatial and single-cell transcriptomic evidence that Bin cell (black) translocation from the B-cell follicles (green) into the sinuses is associated with localization to MARCO^+^ peri-follicular medullary sinuses and IgG2b class-switching as TNF-Tg mice progress from Early (PLN expansion) to Advanced (PLN collapse) arthritis **(A, B)**. As normal lymphatic drainage from Early RA joints **(C)** is known to be lost in Advanced RA associated with lymphatic muscle and endothelial cell death (4, 5, 19), we propose that iron-laden macrophages develop through efferocytosis of these apoptotic cells as macrophages attach to the lymphatic vessels (5) **(D)**. This then supposedly inhibits further ferritin production via negative feedback of *Fth1* gene expression **(E)**. We then propose that the activated and iron-laden macrophages stimulate the recruited T-cells into helper T-cells through interaction of the ALCAM ligand (macrophage) and CD6 receptor (T-cell), identified through bioinformatic analysis and proximity of these cells via IHC in Advanced PLNs **(F)**. These activated T-cells then supposedly stimulate the naïve Bin cells through CD40LG/IL21 (T-cell) and CD40/IL21R (B-cell) pathways **(G)**, which leads to IgG2b^+^ class-switching and plasma cell differentiation, respectively **(H)**. Thus, passive lymphatic flow is then mechanically inhibited as the plasma cells accumulate and clog the sinuses **(I)**. The iron-laden inflammatory macrophages then aggregate within the synovium of afferent joints to promote inflammation, osteoclastogenesis (black arrows), bone resorption, and arthritic flare **(J)**. Our study offers various pieces of evidence to support this novel model of seronegative plasmocytic arthritic flare, which together are provided to guide future investigation towards confirming and elaborating the detailed mechanistic underpinnings. Schematic created on BioRender.com.

Most importantly, the current study also generates new hypotheses regarding the relationship between lymphatic dysfunction and arthritic flare, as illustrated in **Figure 6**, to guide future investigations. During Early arthritis, the B-cells in joint-draining PLNs exhibit the first signs of activation with accumulation of CD21^+^/CD23^+^ Bin-cells. In Early stages of arthritis while the PLN expands in volume, these Bin cells are maintained within the PLN follicle, depicted by black cells amidst green B-cells localized to the follicle within the PLN cortex. At this time, afferent lymphatic flow through popliteal lymphatic vessels (PLVs) is maintained by lymphatic contractions, allowing for high-velocity flow of egressing immune cells through the PLN (**Figure 6A**). However, the onset of Advanced arthritis is associated with reduced PLV contractility and lymph flow related to lymphatic muscle cell (LMC) damage with deficient PLV-LMC coverage. These findings occur concomitant with translocation of Bin cells from the follicle and into the PLN sinuses, supposedly recruited by macrophages now stagnant within the sinuses, leading to further reduction in lymphatic drainage and exacerbation of bone erosions. Associated with the reduced fluid flow, the lymph node reduces in volume, a process termed lymph node collapse. The progression from lymph node expansion to collapse was previously considered to be associated solely with “clogging” of lymphatic flow as anti-CD20 B-cell depletion was able to restore passive lymph drainage, even though PLV contractions remained ineffective. In addition, detailed bulk-tissue investigation of the B-cells within TNF-Tg PLNs was unable to detect changes between the Bin cells located within the follicle during PLN expansion, and those that pathologically translocate into the PLN sinuses during collapse. These proposed mechanisms and investigations have been described in detail through our prior work (4, 5, 10, 12–14, 19, 21, 56–60).

Through utilization of spatial transcriptomics, we were able to target the PLN sinus region during the progression from Early (PLN expansion) to Advanced (PLN collapse) arthritis for specific evaluation of particular cells and gene expression changes involved in B-cell translocation. After more than a decade of investigation, we have determined that the enigmatic migration of B-cells from the PLN follicle and into the sinuses is not solely a passive process. Instead, we identified that the affected sinus region in TNF-Tg mice localizes to the MARCO^+^ sinuses, which have been shown through detailed scRNAseq analysis, to be associated with the peri-follicular medullary region of the PLN (24). Through both transcriptomics and histology evaluation, we further demonstrated that iron-laden macrophages accumulate within these MARCO^+^ sinus regions. In these same PLN regions, CD6^+^ T cells are located in close proximity to ALCAM^+^ macrophages, and our findings suggested these cells interact specifically in Advanced disease. Thus, we propose that CD6/ALCAM co-stimulation leads to IgG2b^+^ class-switching and plasma cell differentiation of the Bin cells that have translocated into the PLN sinuses, recruited by the amassed iron-laden macrophages (**Figure 6B**).

Our study further highlights potential step-by-step mechanisms to explain the puzzling process during which initial lymph node expansion eventually culminates in lymph node collapse as RA progresses. Particularly, we propose that in Early disease these iron-laden macrophages egress from inflamed joints and travel through the PLN with great speed within fully functioning lymphatics (**Figure 6C**), and have no co-stimulatory effects on resident lymphocytes within the PLN. However, with the onset of Advanced arthritis and lymphatic dysfunction, these activated macrophages stagnate within the popliteal lymphatic vessels and PLN, and mediate tissue destruction and immune cell activation (14). This lymphatic stagnation is associated with LEC (5) and LMC (59–63) apoptosis and efferocytosis by joint-draining macrophages leading to intracellular iron accumulation (**Figure 6D**). These iron-laden macrophages then likely exhibit reduced *Fth1* expression through negative feedback (**Figure 6E**), passively flow into the efferent PLN, and exhibit an M1-polarized phenotype (39) with secretion of chemokines (i.e., CCL19) to promote CCR7^+^ T-cell recruitment and B-cell translocation into the sinuses (13). As CCL19 expression was appreciated primarily in the PLN stroma (64), macrophage secretion (65) into the stromal tissue may modify the homeostatic gradient of CCL19 signals to explain the enhanced lymphocyte recruitment to the PLN sinus region of Advanced TNF-Tg mice.

Within this microenvironment, we propose that ALCAM co-stimulation of CD6^+^ T-cells promotes activation and differentiation of the recruited CCR7^+^ T-cells into CD4^+^ helper T-cells (**Figure 6F**). These activated helper T-cells then interact with the Bin cells via CD40LG/IL21 (T-cells) and CD40/IL21R (B-cells) (**Figure 6G**) to promote IgG2b^+^ class-switch recombination and plasma cell differentiation, respectively (**Figure 6H**). Without sufficient lymphatic flow, the IgG2b^+^ plasma cells then accumulate in the MARCO^+^ peri-follicular medullary sinuses, which may physically inhibit passive lymphatic flow *via* clogging of the sinuses and/or produce autoantibodies targeting factors essential for immune cell flow through efferent PLN lymphatics, as described in discussion of **Figures 6A, B** (**Figure 6I**). In this double-hit hypothesis, with the loss of active lymphatic flow by LEC/LMC apoptosis and the inhibition of passive lymphatic flow via plasma cells, the activated macrophages accumulate within the afferent synovium to promote onset of severe inflammatory-erosive arthritis. This model coincides with our findings of increased iron-laden macrophages within the afferent synovium of Advanced TNF-Tg ankle joints, which may then serve as osteoclast precursors, where stagnation within the synovium due to reduced lymphatic flow increases the probability of osteoclastogenesis, especially in a TNF-rich environment (66) (**Figure 6J**). To further evaluate this proposed model, future investigation of *in vivo* cell tracking from the joint to the PLN and relative osteoclastogenic potential of iron-laden macrophages is warranted. Overall, this revised model of lymphatic failure and arthritic flare provides new molecular and cellular targets for future experimentation, and potential insight into the pathogenesis of seronegative RA.

To our knowledge, this effort marks the first attempt to elucidate lymphatic dysfunction during arthritic progression via spatial transcriptomics. However, our study has noteworthy limitations, including: 1) the inability of the approach to detect low level transcripts, protein synthesis, and post-translational modification, 2) the lack of single-cell resolution for spatial transcriptomics, which complicates evaluation of particular histologic domains in cell-dense and heterogenous tissues, and 3) the absence of additional datasets from other RA animal models and human patients with Early and Advanced arthritis to corroborate our results. We also need to evaluate our molecular findings with lymphatic functional defects using the most current noninvasive near-infrared imaging methods (67). Nevertheless, these findings demonstrate the power of spatial transcriptomics, in combination with scRNAseq, to address long-standing questions that could not be resolved by other approaches.

## Data availability statement

The datasets presented in this study can be found in online repositories. The names of the repository/repositories and accession number(s) can be found below: https://www.ncbi.nlm.nih.gov/geo/, GSE195598, GSE211124.

## Ethics statement

The studies involving humans were approved as indicated for each tissue type. Lymph nodes: Collection of specimens from RA autopsy subjects was conducted with written and signed consent from their family members in accordance with the Declaration of Helsinki and after approval from the Ethical Committee of the National Institute of Medical Sciences and Nutrition “Salvador Zubirán”. Control lymph node specimens were collected based on approved protocols by the Committee of Human Research. Analysis of deidentified tissue specimens was performed according to protocols approved by the University of Rochester Institutional Review Board. Synovium: Experiments were approved by the Research Subjects Review Board (RSRB00055411) at the University of Rochester and collected using Accelerating Medicines Partnership (AMP) standard operating procedures. The studies were conducted in accordance with the local legislation and institutional requirements. The participants provided their written informed consent to participate in this study. All animal experiments were approved by the University Committee for Animal Resources at the University of Rochester. The study was conducted in accordance with the local legislation and institutional requirements.

## Author contributions

HK, ES, and CW contributed to 1) the conception or design of the work, 2) acquisition, analysis, or interpretation of the data, and 3) drafted or revised the work. All other authors contributed to 1) the acquisition, analysis, or interpretation of the data and 2) drafted or revised the work. All authors have approved the submitted version of the manuscript. All authors also agree to be personally accountable for their contributions and ensure to appropriately respond to any questions related to the accuracy or integrity of the work. All authors contributed to the article and approved the submitted version.
