# Supplementary figures and images for: Multi-omics analysis identifies IgG2b class-switching with ALCAM-CD6 co-stimulation in joint-draining lymph nodes during advanced inflammatory-erosive arthritis
Source: Front Immunol. 2023 Aug 25;14:1237498. doi: 10.3389/fimmu.2023.1237498 (PMC10485835; doi:10.3389/fimmu.2023.1237498)

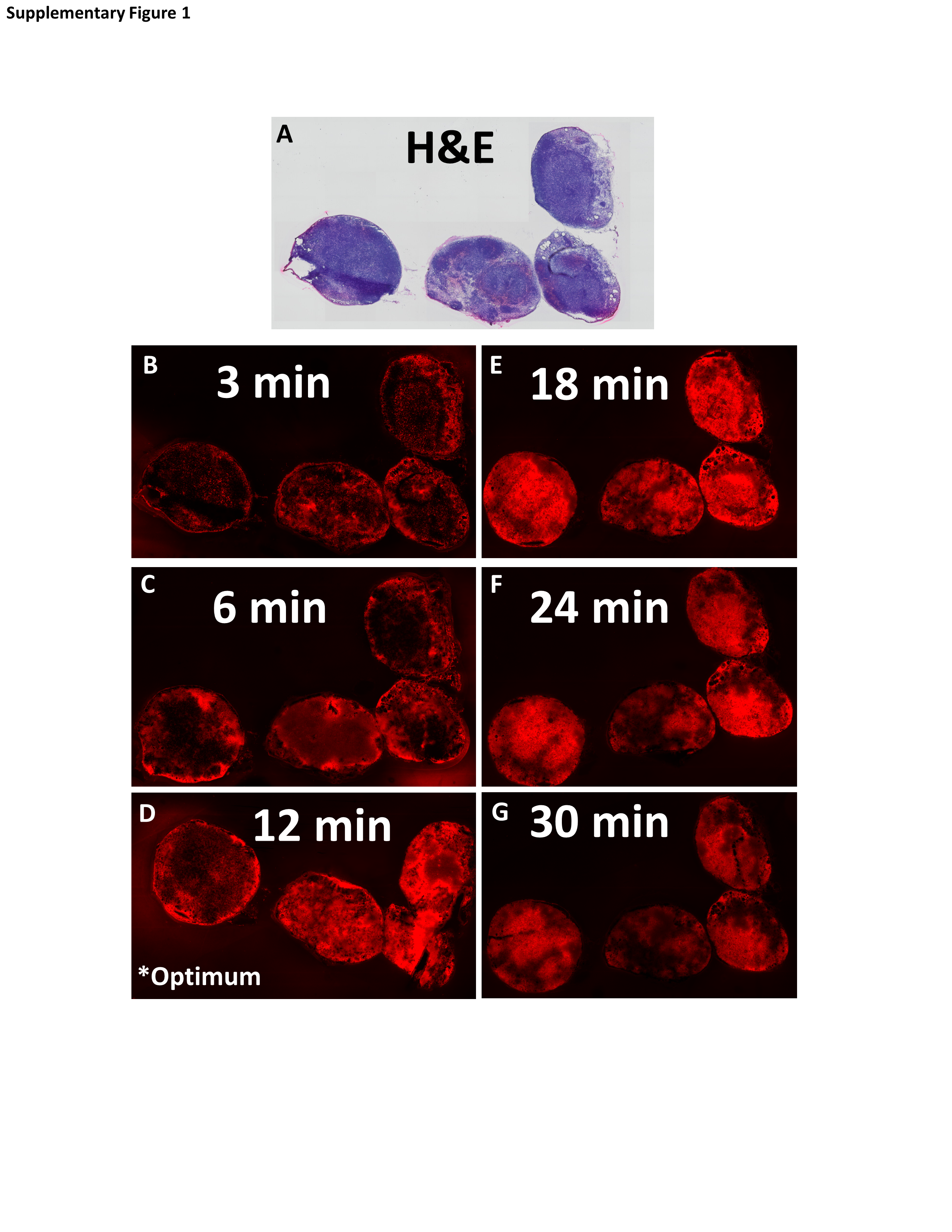

Supplement: Supplementary file 1 [file Image_1.tif]

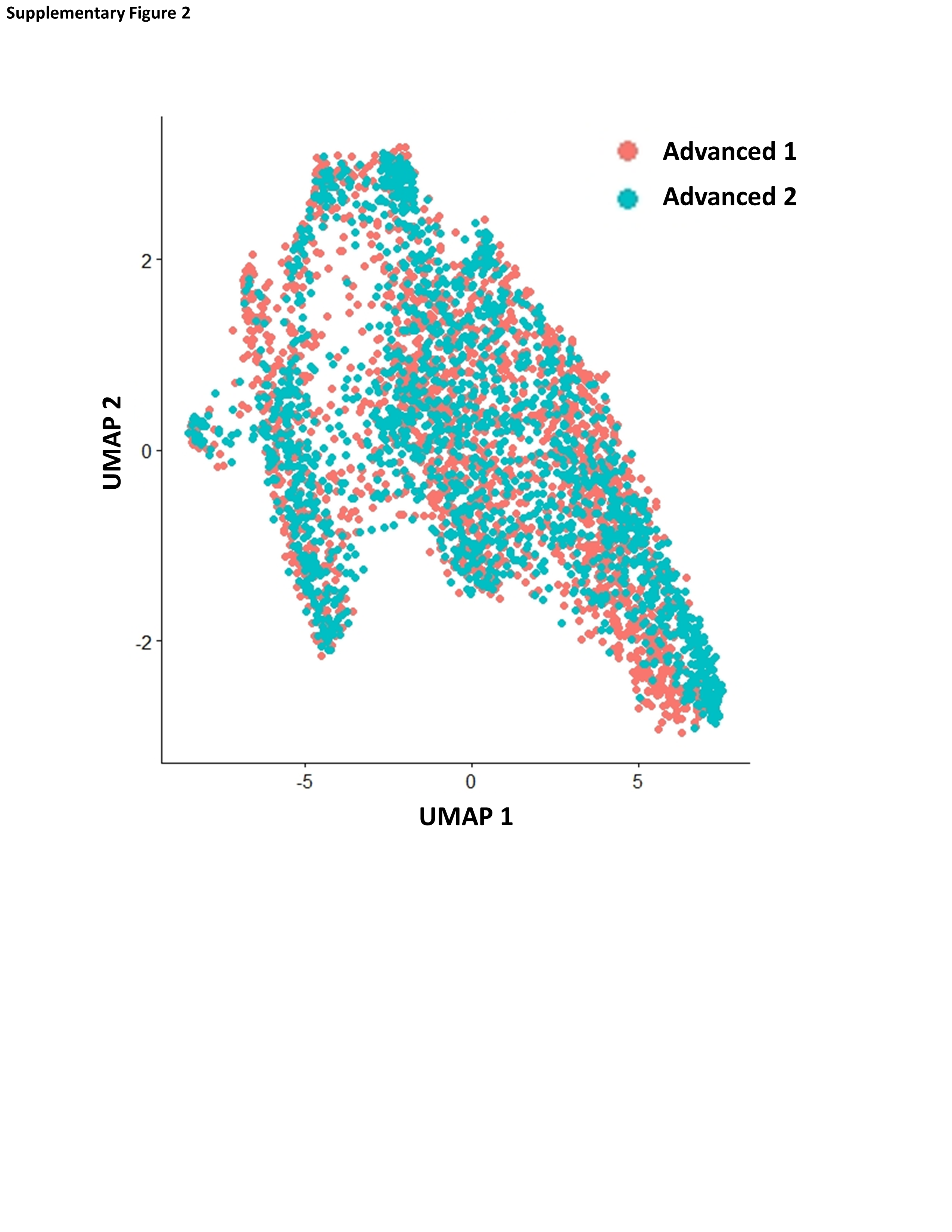

Supplement: Supplementary file 2 [file Image_2.tif]

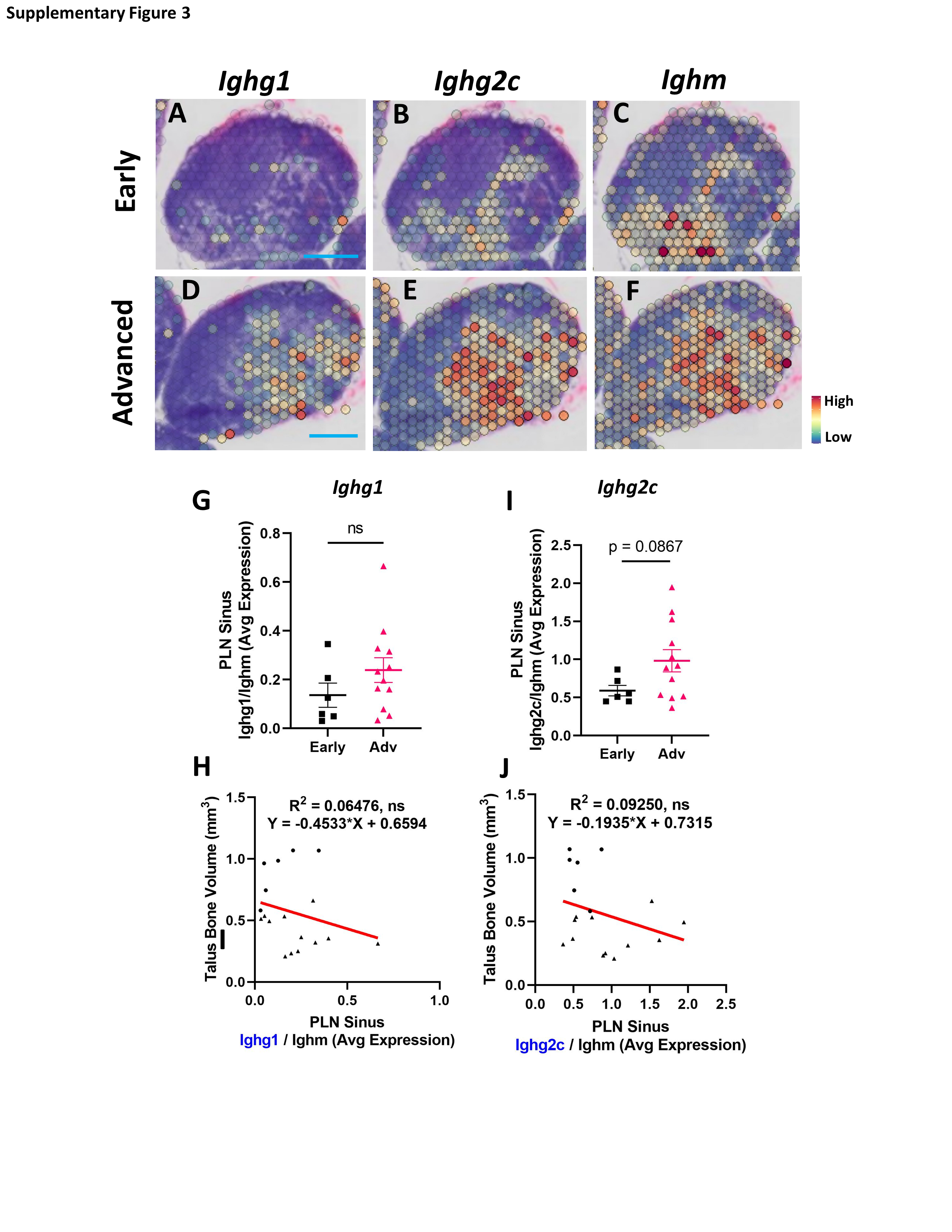

Supplement: Supplementary file 3 [file Image_3.tif]

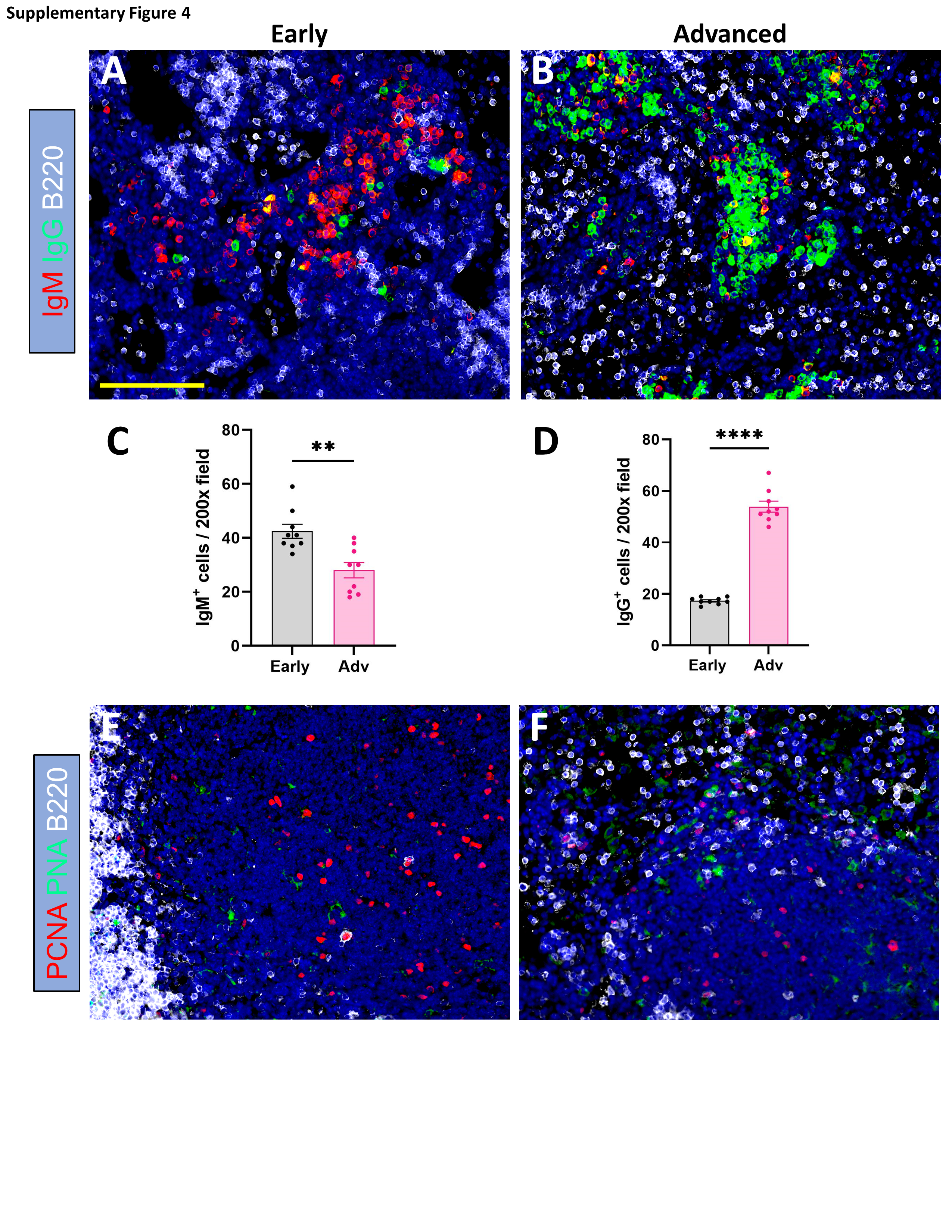

Supplement: Supplementary file 4 [file Image_4.tif]

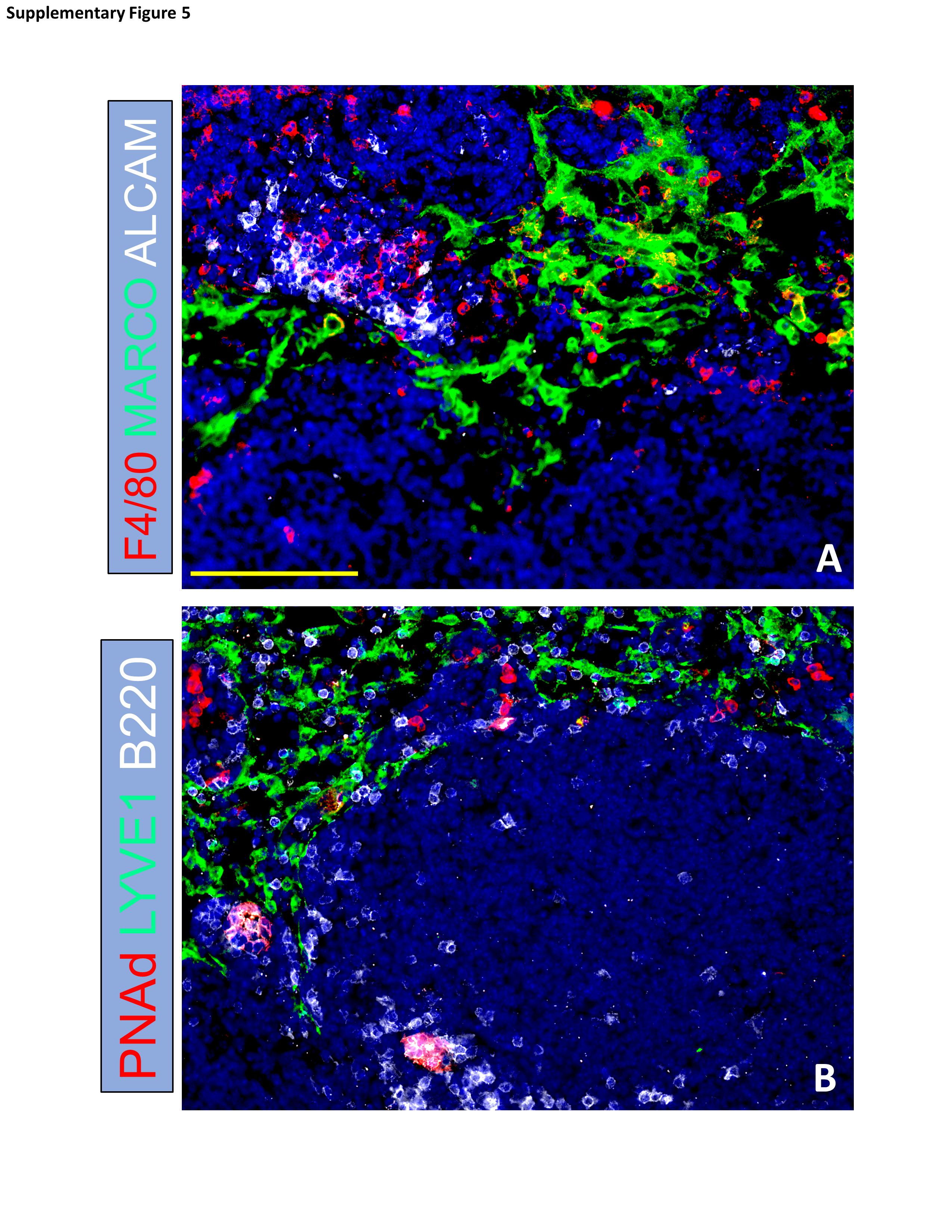

Supplement: Supplementary file 5 [file Image_5.tif]

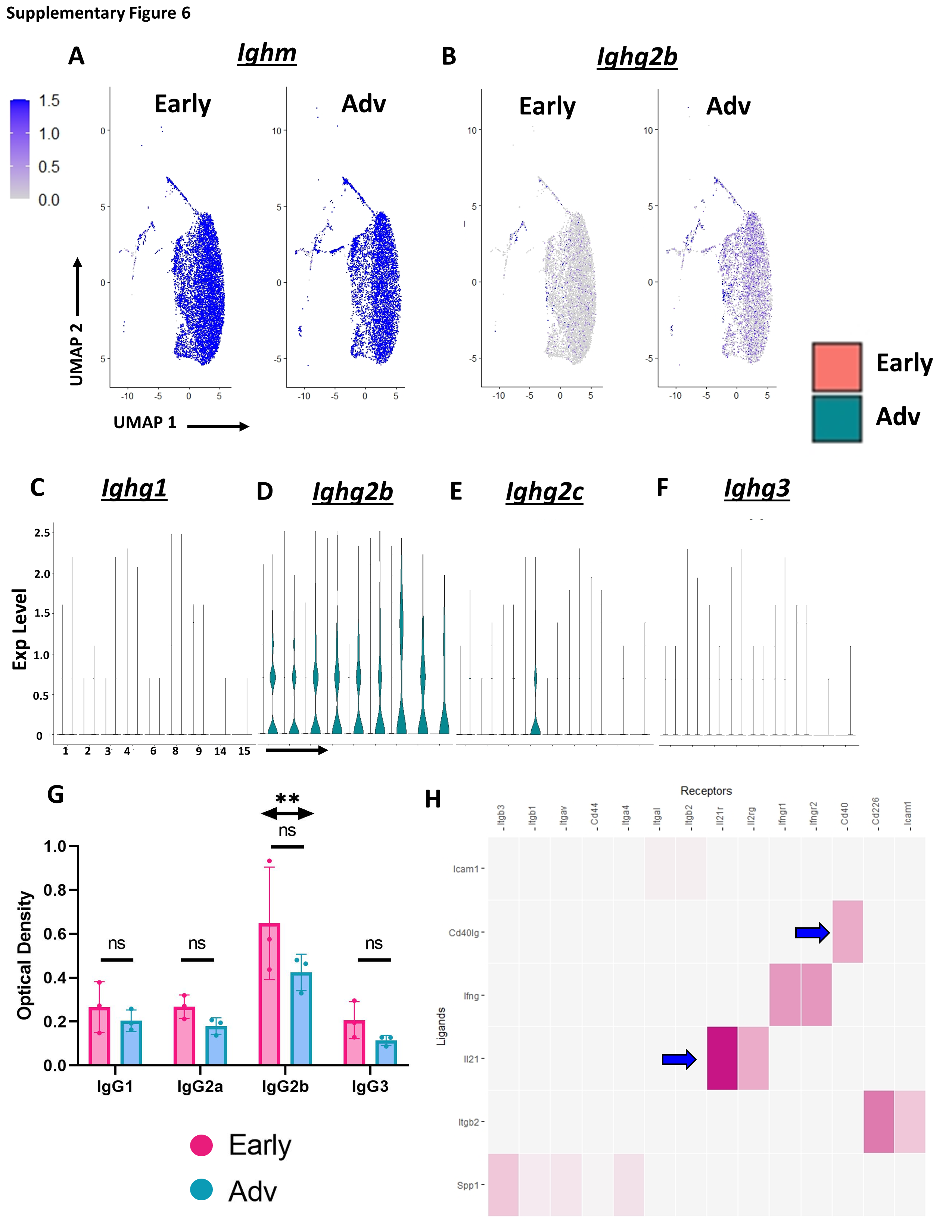

Supplement: Supplementary file 6 [file Image_6.tif]

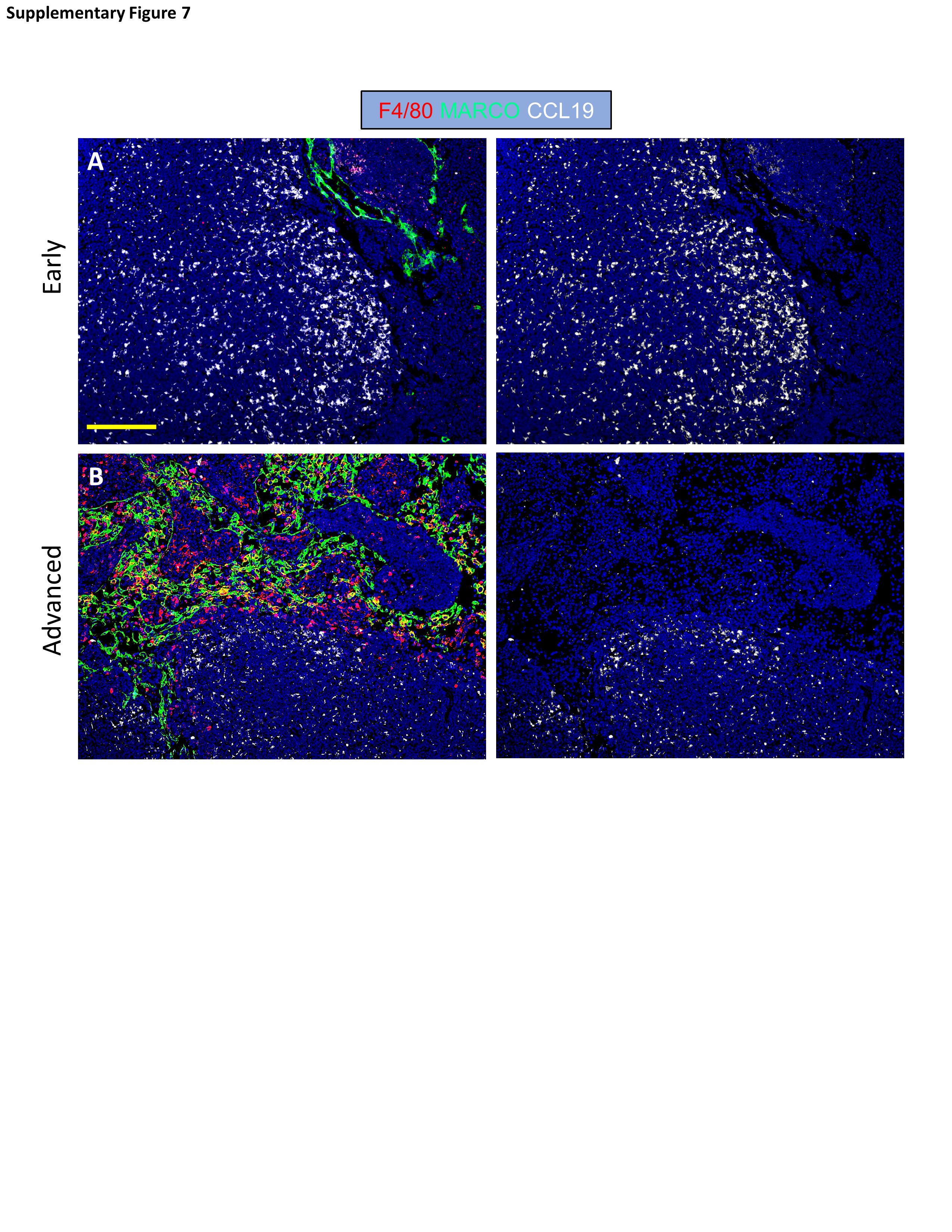

Supplement: Supplementary file 7 [file Image_7.tif]
