# Supplementary material for: Multi-omics analysis identifies IgG2b class-switching with ALCAM-CD6 co-stimulation in joint-draining lymph nodes during advanced inflammatory-erosive arthritis
Source: Front Immunol. 2023 Aug 25;14:1237498. doi: 10.3389/fimmu.2023.1237498 (PMC10485835; doi:10.3389/fimmu.2023.1237498)
Supplement: Supplementary file 8 [file Table_1.docx]

| **Sample** | **RNA Integrity Numbers (RIN)** |
| --- | --- |
| Optimization | 9.4 |
| Wild-Type | 10.0 |
| TNF-Tg Early | 9.6 |
| TNF-Tg Advanced 1 | 9.6 |
| TNF-Tg Advanced 2  **Supplementary Table 1** | 9.6 |
