# Supplementary material for: Multi-omics analysis identifies IgG2b class-switching with ALCAM-CD6 co-stimulation in joint-draining lymph nodes during advanced inflammatory-erosive arthritis
Source: Front Immunol. 2023 Aug 25;14:1237498. doi: 10.3389/fimmu.2023.1237498 (PMC10485835; doi:10.3389/fimmu.2023.1237498)
Supplement: Supplementary file 9 [file Table_2.docx]

| **Cluster #** | **Cluster Identity** | **Early (%)** | **Advanced (%)** |
| --- | --- | --- | --- |
| 1 | CD55+ / Ighd+ / Cd21+ / Cd23+ Bin Cells | 32.45% | 26.62% |
| 2 | Ly6d+ / Mzb1+ Pre B1 Cells | 34.90% | 22.85% |
| 3 | Ighd+ / Vpreb3+ Naïve B-Cells | 9.13% | 9.08% |
| 4 | Ahr+ / Zbtb20+ / Mzb1+ / CD24a+  Activated B1 Cells | 9.11% | 6.00% |
| 5 | Ccr7+ / Cd27+ / Cd96+ / Cd226+  Activated T-Cells | 0.50% | 8.60% |
| 6 | Nme1/2+ Class-Switching B-Cells | 4.90% | 3.44% |
| 7 | Cd4+ / Cd8- / Cd40lg+  Activated Helper T-Cells | 1.32% | 4.14% |
| 8 | Nme1/2+ / Mki67+ / Top2a+ / Jchain+ Proliferative Class-Switching B-Cells | 2.08% | 2.97% |
| 9 | Sec61a1+ Activated B-Cells | 1.65% | 3.18% |
| 10 | Hfe+ / Cx3cr1+ / Cd88+ / Aif1+  Inflammatory Macrophages | 1.54% | 2.56% |
| 11 | Cd4- / Cd8+ / Ly6c2+ / Cd160+ / Cxcr3+ Activated Cytotoxic T-Cells | 0.21% | 3.09% |
| 12 | Tmem176a+ / Cx3cr1+  Immature Dendritic Cells | 0.77% | 2.36% |
| 13 | Ly6c2+ / Ccr2+ / Itgax+ Monocytes | 0.74% | 1.51% |
| **14** | **Cd93+ / Irf4+ / Cxcr4+ Plasma Cells** | **0.07%** | **1.52%** |
| 15 | Dntt+ / Sox4+ Pro-Lymphocytes | 0.15% | 1.11% |
| 16 | Hfe- / Cd86+ / Vcam1+ / Aif1+  Inflammatory Macrophages | 0.34% | 0.71% |
| 17 | Kit+ / Mcpt4+ / Cma1+ Mast Cells | 0.35% | 0.58% |
| 18 | Cd207+ / Cd8+ Dendritic Cells | 0.16% | 0.25% |

**Supplementary Table 2**
