# Supplementary material for: Multi-omics analysis identifies IgG2b class-switching with ALCAM-CD6 co-stimulation in joint-draining lymph nodes during advanced inflammatory-erosive arthritis
Source: Front Immunol. 2023 Aug 25;14:1237498. doi: 10.3389/fimmu.2023.1237498 (PMC10485835; doi:10.3389/fimmu.2023.1237498)
Supplement: Supplementary file 13 [file DataSheet_1.docx]

**Supplementary Figures**

**Multi-omics Analysis Identifies IgG2b Class-Switching with ALCAM-CD6 Co-Stimulation in Joint-Draining Lymph Nodes During Advanced Inflammatory-Erosive Arthritis**

H. Mark Kenney PhD^1,2^, Javier Rangel-Moreno PhD^3^, Yue Peng MS^1,2^, Kiana L. Chen MS^1,2^, Jennifer Bruno BS^4,5^, Abdul Embong MS^5^, Elizabeth Pritchett PhD^6^, Jeffrey I. Fox BS^1^, Enrique Becerril-Villanueva PhD^11^, Armando Gamboa-Domínguez PhD^12^, Sally Quataert PhD^4,5^, Gowrishankar Muthukrishnan PhD^1,5,7^, Ronald W. Wood PhD^1,8,9,10^, Benjamin D. Korman MD^1,3^, Jennifer H. Anolik MD PhD^1,2,3^, Lianping Xing PhD^1,2^, Christopher T. Ritchlin MD MPH^1,3^, Edward M. Schwarz PhD^1,2,3,5,7,10,*^, and Chia-Lung Wu PhD^1,7^

^1^Center for Musculoskeletal Research; ^2^Department of Pathology & Laboratory Medicine; ^3^Department of Medicine, Division of Allergy, Immunology, Rheumatology; ^4^Center for Vaccine Biology and Immunology; ^5^Department of Microbiology and Immunology; ^6^Genomics Research Center; ^7^Department of Orthopaedics, ^8^Department of Obstetrics and Gynecology, ^9^Department of Neuroscience, ^10^Department of Urology, University of Rochester Medical Center, Rochester NY, USA

^11^Psychoimmunology Laboratory, Instituto Nacional de Psiquiatría "Ramón de la Fuente Muñiz", Mexico City, Mexico

^12^Department of Pathology, Instituto Nacional de Ciencias Médicas y Nutrición Salvador Zubirán, Mexico City, Mexico

*Corresponding Author; Edward_Schwarz@URMC.Rochester.edu; (585) 276-5609; University of Rochester Medical Center, 601 Elmwood Ave, Box 665, Rochester, NY, 14642, USA.

**Supplementary Figure 1. Optimization of tissue permeabilization for PLNs.** PLNs from WT mice (n=2 mice, 4 PLNs) were harvested and processed to determine the optimal incubation for tissue permeabilization. A representative H&E-stained section is shown **(A)** along with representative images of TRITC-fluorescently tagged nucleotides after 3-minutes **(B)**, 6-minutes **(C)**, 12-minutes **(D)**, 18-minutes **(E)**, 24-minutes **(F)**, and 30-minutes **(G)** of tissue permeabilization. 12-minutes was the optimal time for permeabilization based on signal intensity and limited diffusion.

**Supplementary Figure 2. Integration of Advanced capture areas confirms consistency of the replicates.** We performed CCA integration of both Advanced capture areas and evaluated the consistency of the principal components of the two replicates embedded on a UMAP. Based on the direct overlay and lack of unique spot populations between Advanced 1 vs Advanced 2, we considered the Advanced capture areas to be replicates. Thus, the Advanced capture areas were merged together as a single group for comparison with the WT and Early capture areas.

**Supplementary Figure 3. PLN immunoglobulin expression and relationship with arthritic severity is selective for *Ighg2b*.** Corresponding with the immunoglobulin gene expression analysis and talus bone volumes in Figure 3, we also evaluated the levels of *Ighg1* and *Ighg2c* relative to *Ighm*. The expression of these genes is shown as a representative spatial feature plot overlaying H&E stained PLNs in the Early and Advanced condition **(A-F**, high-magnification images from Figs 1B&D [rotated]**)**. In contrast to *Ighg2b/Ighm* (Figure 3), neither *Ighg1/Ighm* nor *Ighg2c/Ighm* expression is increased in Advanced PLN sinuses. Additionally, unlike *Ighg2b/Ighm* and *Ighg3/Ighm,* the expression of *Ighg1/Ighm* and *Ighg2c/Ighm* does not correlate with talus bone volumes in the afferent ankles **(G-J)**. *Ighg2a* and *Ighg4* were omitted as these genes were not expressed within the datasets. Blue scale bar = 0.5mm **(A-F)**.

**Supplementary Figure 4. IgM to IgG conversion without change in B-cell proliferation in Advanced PLNs.** Relative to Early PLNs with abundant IgM^+^ cells (red) **(A)**, Advanced PLNs exhibited a predominance of IgG^+^ (green) cells **(B)**. Quantitatively, Early PLNs had significantly increased IgM^+^ cells compared to Advanced **(C)**, while Advanced PLNs showed significantly increased IgG^+^ cells **(D)**. The increased IgG^+^ cells in Advanced PLNs was also not associated with a corresponding change in cell proliferation measured by proliferating cell nuclear antigen (PCNA, red), which suggests that the IgG^+^ cells represent mature non-proliferating plasma cells. Peanut agglutinin (PNA, green) marks B-cells that may associate with germinal centers **(E&F)**. Statistics: Unpaired t-test **(C&D)**, ***p<0.01*, *****p<0.0001*. Yellow scale bar = 100μm **(A&B, E&F)**.

**Supplementary Figure 5. ALCAM^+^ and F4/80^+^ macrophages localize to MARCO^+^/LYVE1^+^ lymphatic endothelial cell lined sinuses.** In a representative Advanced PLN, immunofluorescence revealed ALCAM^+^ (white) / F4/80^+^ (red) macrophages closely localized to the MARCO^+^ (green) PLN sinuses **(A)**. LYVE1 (green) immunostaining was demonstrated in the same cellular pattern and location within the PLN sinuses as the MARCO^+^ cells in **A**, further confirming the MARCO^+^ lymphatic endothelial cell identity. On the other hand, peripheral node addressin (PNAd, red) marks high endothelial venules **(B)**. Note, as the F4/80^+^ cells localized to similar regions of the PLN as the iron-laden cells, and because macrophages are known to be the primary immune cell regulator of iron homeostasis (1), the iron-laden cells are presumed to be macrophages. Yellow scale bar = 100μm **(A&B)**.

**Supplementary Figure 6. B-cell immunoglobulin expression is selective for *Ighg2b* in Advanced PLNs.** To validate the increased *Ighg2b* expression noted within the PLNs of TNF-Tg mice with Advanced arthritis by spatial transcriptomics, we evaluated the immunoglobulin isotypes between the Early and Advanced conditions after subsetting the B-cell populations by scRNAseq. High gene expression of *Ighm* was noted within all B-cell subtypes regardless of arthritic severity **(A)**, while Advanced B-cells showed increased *Ighg2b* levels compared to Early **(B)**. The increased immunoglobulin expression in Advanced B-cells was selective to the *Ighg2b* isotype with limited expression in both Early and Advanced conditions for *Ighg1*, *Ighg2c*, and *Ighg3* **(C-F)**. *Ighg2a* and *Ighg4* were undetectable throughout the datasets, and are thus omitted. Interestingly, despite the increased *Ighg2b* expression in Advanced PLNs, there was no difference in serum IgG2b antibody levels between Early and Advanced TNF-Tg mice. Both TNF-Tg cohorts exhibited expected proportions of immunoglobulin isotypes with IgG2b as the predominant isotype in C57BL/6 mice (2) **(G)**. Towards understanding the IgG2b^+^ plasma cell accumulation in PLNs, interaction analysis between *Cd4^+^/Cd8^-^/Cd40lg^+^* activated helper T-cells (ligands) and *Nme1/2^+^/Mki67^+^/Top2a^+^/Jchain^+^* proliferative class-switching B-cells (receptors) (blue stars as in Figure 5A) showed high interaction potential with *Cd40lg* / *Il21* (T-cells) and *Cd40* /*Il21r* (B-cells) as plausible cellular mechanisms of IgG2b class-switching and plasma cell differentiation, respectively **(H**, blue arrows).

**Supplementary Figure 7. Detection of CCL19 expression in TNF-Tg PLNs**. Both Early **(A)** and Advanced **(B)** PLNs demonstrated expression of CCL19 (white), especially by F4/80^-^/MARCO^-^ (red/green) cells in the stroma (composite left, CCL19 alone right panel). However, similar to Figure 4, the number of macrophages (F4/80^+^ and/or MARCO^+^ cells) may be increased in the PLN sinuses during Advanced arthritis. The changes in cellular composition may modify the CCL19 distribution within the lymph node architecture and explain enhanced lymphocyte recruitment to the MARCO^+^ sinus region during arthritic progression. In addition, as CCL19 is expressed by F4/80^-^/MARCO^-^ cells in the stroma, multiple cell types are likely involved in the dynamic cellular changes in inflamed PLNs. Yellow scale bar = 100μm **(A&B)**.

**Supplementary Table 1. RIN values for estimating RNA integrity.** Prior to utilization of the PLN tissue blocks for analysis, ten-consecutive 10μm sections were collected to evaluate RNA quality through RIN quantification. We considered RIN values >8.0 to be optimal for gene expression analysis, and all tissue showed RIN values in this range with an overall minimum RIN value of 9.4 (Optimization). There was a minimum RIN value of 9.6 for the gene expression blocks (all TNF-Tg), and a maximum RIN value of 10.0 (WT), which is the highest possible RIN value.

**Supplementary Table 2. Single-cell cluster identity.** Corresponding with the 18 clusters shown by UMAP (Figure 5), the complete cluster identities and proportion of total cells for the Early and Advanced conditions are provided in this table. The complete cluster identities provide the associated genes and annotations of each cluster number. Rows are highlighted by color corresponding with the populations used for cell-cell interaction analysis as in Figure 5D (yellow) and Supplementary Figure 6H (blue), while plasma cells are identified by bolded text.

**Supplementary Videos.** The videos demonstrate a fly-through of an image-stack from the original Prussian Blue and Nuclear Fast Red stained images. Note the rotation artifact of the images, which is a product of the alignment procedures performed in Amira software. Color segmentation was performed in ImageJ using the “Color Deconvolution 2” plug-in with a threshold set on the “Feulgen LightGreen” vector. The resultant segmentations were imported into Amira, aligned with the histology, and rendered in 3D to depict the lymph node stromal cells (red/orange) and the iron-laden cells within the sinuses (blue). The video animations were created in Amira software.

**References**

1. DeRosa A, Leftin A. The Iron Curtain: Macrophages at the Interface of Systemic and Microenvironmental Iron Metabolism and Immune Response in Cancer. *Front Immunol* (2021) 12(614294). doi: 10.3389/fimmu.2021.614294.

2. Klein-Schneegans A, Kuntz L, Fonteneau P, Loor F. Serum concentrations of IgM, IgG1, IgG2b, IgG3 and IgA in C57BL/6 mice and their congenics at the lpr (lymphoproliferation) locus. *J Autoimmun* (1989) 2(6):869-75. doi: 10.1016/0896-8411(89)90013-9.
